# Herpes simplex virus pUL56 abolishes neuronal activity by removing voltage-gated ion channels from the plasma membrane

**DOI:** 10.1126/sciadv.aei1796

**Published:** 2026-10-07

**Authors:** Daniel A. Nash, Henry G. Barrow, P. Robin Antrobus, Alex S. Nicholson, John Suberu, Harvey E. Johnston, Martin Potts, Marta A. Almeida, Valeria Lulla, Colin M. Crump, Anton J. Enright, Michael P. Weekes, Janet E. Deane, Stephen C. Graham

**Affiliations:** ^1^ Department of Pathology, University of Cambridge, Cambridge CB2 1QP, UK.; ^2^ Cambridge Institute for Medical Research, University of Cambridge, Cambridge CB2 0XY, UK.

## Abstract

Herpes simplex virus 1 (HSV-1) infections of the central nervous system cause encephalitis and are associated with increased risk of neurodegeneration, yet the molecular consequences of lytic infection in human neurons remain incompletely defined. We map the transcriptomic, proteomic, and surface-proteome changes induced by HSV-1 across the lytic infection cycle in human induced pluripotent stem cell–derived cortical glutamatergic neurons. HSV-1 drives extensive plasma-membrane remodeling, including the removal of voltage-gated sodium, potassium, and calcium channels, resulting in a profound loss of synchronous calcium signaling. We identify the viral ubiquitin-ligase adaptor pUL56 as the principal effector of this process: pUL56-dependent degradation of ion channels abolishes coordinated calcium signaling, whereas mutation of its E3-ligase–binding motifs preserves synchrony. Furthermore, expression of pUL56 alone is sufficient to abolish neuronal electrical activity. These findings establish pUL56 as a potent viral suppressor of neuronal excitability.

## INTRODUCTION

Approximately 65% of people under the age of 50 are infected with herpes simplex virus 1 (HSV-1) (*1*). HSV-1 infections generally start in peripheral tissues, particularly fibroblasts or keratinocytes of the orofacial or genital mucosa. The virus replicates in these peripheral tissues before infecting sensory or sympathetic neurons, enabling spread to the trigeminal or superior cervical ganglia where they establish a lifelong latent infection (*2*). The common clinical manifestations of HSV-1 reactivation are cold sores or genital blisters. However, during primary infection or reactivation of latent virus from the trigeminal ganglia, HSV-1 can spread to the central nervous system (CNS) to cause herpes simplex encephalitis (HSE), a necrotizing hemorrhagic inflammatory encephalitis that manifests in the temporal lobes and adjacent limbic tissue (*3*, *4*). HSV-1 infection is the most frequently diagnosed cause of sporadic encephalitis (*5*), affecting approximately one in 250,000 individuals per year (*6*, *7*). While prompt treatment with the antiviral drug acyclovir reduces mortality rates from 70 to less than 20% (*6*, *8*), approximately 50% of HSE survivors experience substantial neurological sequelae (*9*–*11*). Furthermore, HSV-1 infection is linked to increased risk of Alzheimer’s disease (AD) and related dementias (*12*–*15*), and infection studies using human induced pluripotent stem cell (iPSC)–derived three-dimensional models recapitulate multiple AD-like pathologies including amyloid fibrils, neuroinflammation, and diminished neural network functionality (*16*).

Much is known about the molecular consequences of lytic HSV-1 infection in peripheral cells (*17*–*19*), and there is substantial literature describing infection plus mechanisms of latency in sensory neurons (*20*, *21*). However, much less is known about the molecular consequences of lytic HSV-1 infection in cells of the human CNS. Animal models enable the study of HSV-1 infection pathology (*22*), and organoid models represent a promising system for analyzing transcriptional changes across different human cell types (*23*). However, these models are not currently amenable to high-resolution proteomic analysis, for example, of subcellular protein organization. Recent advances in iPSC technology allow rapid differentiation of specific neuronal subtypes, including sensory or cortical neurons (CNs) following expression of NGN3 or NGN2, respectively (*24*–*26*). These iPSC systems complement more complex models by allowing highly efficient infection in an isogenic background (*27*–*29*) and are scalable to allow unbiased screening approaches like whole-cell and subcellular proteomics (*30*, *31*) and forward genetic screening (*24*).

Quantitative temporal viromics (QTV) is a powerful technique for identifying how viruses remodel the environment of infected cells over the course of an acute infection (*32*). Coupled with transcriptomic analysis, it allows robust identification of cellular proteins that are specifically degraded by virus-encoded proteins in the absence of mRNA degradation, highlighting viral countermeasures against host cell innate or intrinsic immunity (*33*). QTV analysis of human keratinocytes infected with HSV-1 identified that viral protein pUL56 is an adaptor for cellular ubiquitin E3 ligases and, combined with plasma membrane (PM) proteomics, identified that pUL56 stimulates degradation of the cellular trafficking factor GOPC, leading to the down-regulation of the pattern recognition receptor TLR2 from the surface of infected cells (*17*).

Recently, we established protocols for highly efficient synchronous infection of human iPSC–derived cortical glutamatergic neurons (i3Neurons), a relevant neuronal subtype for studying encephalitis (*27*). Here, we perform QTV on HSV-1–infected i3Neurons. We show that voltage-gated ion channels are lost from the surface of infected neurons, abolishing their electrical activity, and this remodeling of the PM proteome is driven by the HSV-1 ubiquitin E3 ligase adaptor pUL56.

## RESULTS

### QTV analysis of HSV-1–infected i3Neurons

The replication kinetics of HSV-1 strain KOS in differentiated i3Neurons was monitored to identify suitable sampling times postinfection for QTV analysis (Fig. 1A). HSV-1 replication was slower than in peripheral cells (*17*), with progeny virus production commencing by 9 hours postinfection (hpi) and peak titers obtained at 30 hpi, indicating completion of the replication cycle. QTV was thus performed by synchronously infecting i3Neurons [multiplicity of infection (MOI) 5] and collecting lysates at 3, 6, 12, 18, and 30 hpi (Fig. 1B). Mock-infected samples were collected at 12 hpi, with additional mock-infected samples collected at 3 and 30 hpi for transcriptomic analyses. Protein and RNA extracts were prepared for proteomic and transcriptomic analysis, with changes to protein abundance quantified using quantitative MS3 mass spectrometry (MS) of 18-plex tandem mass tag (TMT)–labeled samples and using Oxford Nanopore sequencing of cDNA to monitor changes in transcript abundance. Parallel infections confirmed near-100% infection efficiency (fig. S1), with both proteomic (fig. S2A) and transcriptomic data (fig. S3) clustering by infection status and time postinfection rather than by biological replicates. The final analysis quantitated 6135 proteins, of which 61 were viral, and 12,214 transcripts, including 72 viral genes, providing a comprehensive view of proteome and transcriptome changes during HSV-1 infection. A total of 1446 of the proteins identified by MS were not identified in the previous proteomic study of HSV-1–infected keratinocytes (*17*), and many are likely to be neuron specific. Both datasets can be interrogated using an interactive “plotter” worksheet in data S1.

**Fig. 1.**
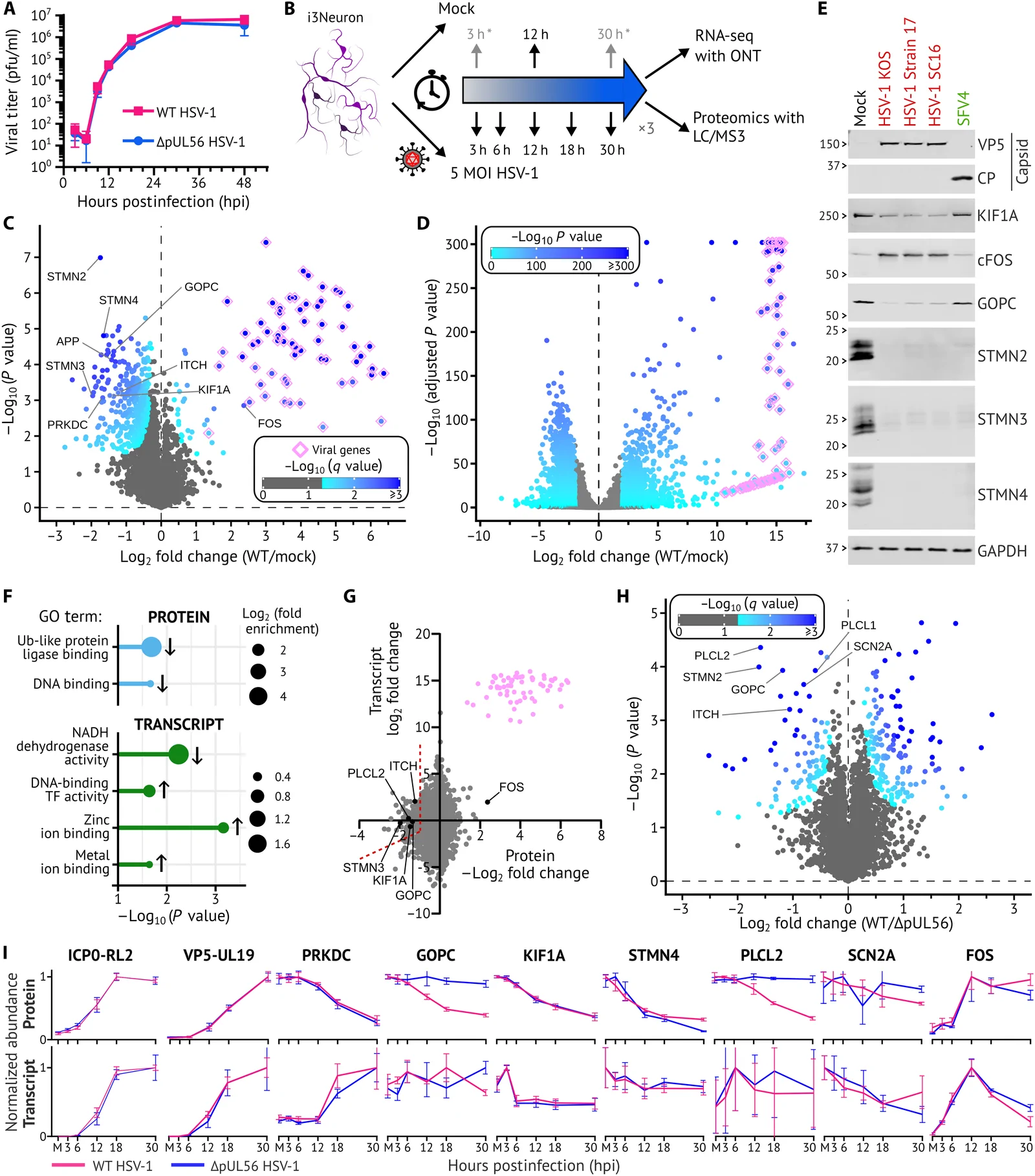
Quantitative temporal viromics of HSV-1 infection in cortical glutamatergic neurons. (**A**) Single-step (MOI 5) replication curve of WT and ΔpUL56 HSV-1 in day 14 i3Neurons. Means ± SD for three independent experiments is shown. (**B**) Schematic of the QTV workflow using day 14 i3Neurons. Mock samples were harvested at 12 hpi for all analyses, and additionally at 3 and 30 hpi for transcriptomics (RNA sequencing) using Oxford Nanopore Technology (ONT). h, hours. (**C**) Changes in i3Neuron protein abundance at 30 hpi with WT HSV-1. Horizontal axis shows average fold change and vertical axis shows significance (two-sided *t* test) for three independent experiments. Proteins are colored by false discovery rate (FDR)–corrected significance and viral proteins are outlined in pink. (**D**) Changes in i3Neuron transcript abundance at 30 hpi with WT HSV-1. Horizontal axis shows average fold change and vertical axis shows FDR-adjusted significance for three independent experiments. Significantly altered genes (log_2_ fold change ≥2 and *P* ≤ 0.05) are blue and viral transcripts are outlined in pink. (**E**) Immunoblot analysis of day 14 i3Neurons at 16 hpi (MOI 10) with HSV-1 strains KOS, S17, and SC16, or with SFV4. Changes in KIF1A, cFOS, and GOPC are HSV-1 specific, whereas abundance of stathmins (STMN2–4) is altered in all infections. Capsid proteins VP5 (HSV) and CP (SFV4) confirm successful infection and glyceraldehyde-3-phosphate dehydrogenase (GAPDH) is a loading control. (**F**) GO analysis of proteins (top, blue) and transcripts (bottom, green) with significantly changed abundance at 30 hpi in HSV- versus mock-infected i3Neurons. Significant GO molecular function terms are shown as bubbles sized by magnitude of enrichment, with significance (FDR-adjusted *P* value) plotted on the horizontal axis and arrows indicating whether the term was enriched amongst the up- or down-regulated gene products. (**G**) Changes in protein (horizontal) versus transcript (vertical) abundance in mock- versus HSV-infected i3Neurons at 30 hpi. Cellular and viral gene products are gray and pink, respectively, with selected cellular gene products highlighted (black). Potential targets of HSV-1 directed proteasomal degradation (larger fold change in protein versus transcript abundance) are to the left of the dotted red line. (**H**) Cellular protein abundance changes in day 14 i3Neurons infected (MOI 5) with WT versus ΔpUL56 i3Neurons. Data from three independent experiments are plotted as in (C). (**I**) Normalized abundance traces of selected proteins (top) and transcripts (bottom) across the time course of infection with WT (pink) or ΔpUL56 (blue) HSV-1. Mock-infected samples (M) are shown as 0 hpi.

As anticipated, the most pronounced changes in proteome composition were observed at 30 hpi (fig. S4A). HSV-1 infection resulted in an overall decrease in cellular protein abundance (observable as a leftward skew in Fig. 1C and data S2) and a significant decrease in the abundance of known HSV-1 targets like PRKDC and GOPC (Fig. 1C), consistent with previous proteomic analysis of keratinocytes (*17*). The stathmin family proteins STMN2 and STMN4 that are expressed predominantly in the brain (*34*, *35*) were also highly down-regulated. In total, 411 proteins were significantly down-regulated by HSV-1 at 30 hpi, with 112 being less than half as abundant as in mock infection, and 86 proteins were significantly up-regulated, of which 61 were viral. cFOS was the most up-regulated cellular protein, likely due to mRNA stabilization following HSV-1 infection (*36*, *37*). Transcriptomic changes were also most pronounced at 30 hpi (fig. S4B), with 1715 transcripts significantly down-regulated and 1322 (including 72 viral transcripts) significantly up-regulated (Fig. 1D and data S3). To validate the proteomics and probe whether abundance changes represent a specific response to HSV-1 infection, immunoblots were performed on lysates prepared from i3Neurons infected for 24 hours at MOI 10 with HSV-1 strains KOS, S17, or SC16, or with the neurotropic RNA virus Semliki Forest virus 4 (SFV4). KIF1A and GOPC were degraded only upon infection with HSV-1, whereas stathmin family proteins STMN2, STMN3, and STMN4 were all decreased following infection with either HSV-1 or SFV4, suggesting that their degradation represents a general neuronal response to virus infection (Fig. 1E). Gene Ontology (GO) analysis of gene products significantly changed in abundance at 30 hpi (Fig. 1F) revealed no molecular function terms enriched among up-regulated proteins, but “ubiquitin-like protein ligase binding” was enriched among down-regulated proteins. Among down-regulated transcripts, “NADH dehydrogenase activity” was significantly enriched, and three other terms related to DNA binding or metal ion–binding proteins were enriched among up-regulated transcripts. There was no significant enrichment of interferon-stimulated genes among the up-regulated proteins or transcripts at any time point.

Hijacking the host ubiquitin ligase axis is crucial during HSV-1 infection; viral proteins that have ubiquitin ligase activity, or that act as ligase adaptor proteins, target host proteins for degradation to counteract innate immune responses (*17*, *38*–*41*). Proteins specifically targeted for ubiquitination and degradation in i3Neurons could thus represent previously unidentified neuronal antiviral restriction factors that HSV-1 can overcome. To identify proteins that are actively degraded, the log_2_ fold change in protein abundance between wild-type (WT) and mock infection was compared with the change in transcript abundance (Fig. 1G). Proteins such as GOPC and ITCH, which are targeted for ubiquitin-mediated proteasomal degradation by HSV-1 pUL56, were degraded despite their transcript levels remaining steady or increasing. Given the known role of pUL56 target protein GOPC in regulating trafficking of AMPA receptors (*42*), the contribution of pUL56 to HSV-1 infection of i3Neurons was investigated. The replication kinetics of HSV-1 lacking pUL56 (ΔpUL56) in i3Neurons was indistinguishable from the WT virus (Fig. 1A), as previously observed for keratinocytes (*17*, *43*). QTV of ΔpUL56 HSV-1 infection was thus performed using the same time points as for the WT virus. A total of 6551 proteins were identified, 89.6% of which were also identified in the WT virus infection (fig. S5). Again, replicates clustered by time postinfection rather than by biological replicates (figs. S2B and S3). Furthermore, the clustering analysis suggests that the transcriptomes of i3Neurons infected with WT and ΔpUL56 HSV-1 are highly similar (fig. S3), and at 30 hpi there were no robust differences in how either virus alters the transcriptome (fig. S6). In contrast, there were significant changes in how WT and ΔpUL56 HSV-1 alter the proteome of infected i3Neurons (Fig. 1, H and I). For example, the abundance of membrane-associated proteins PLCL1, PLCL2, and Na_V_1.2 (SCN2A) that modulate neuronal signaling (*44*–*46*) were reduced at 30 hpi with WT HSV-1 but significantly rescued following infection with HSV-1 lacking pUL56.

### pUL56 targets voltage-gated ion channels for removal from the PM and selective proteolysis

Given the importance of the cell surface in mediating communication between neurons, proteomic analysis was performed to identify whether pUL56 affects the surface abundance of Na_V_1.2 (SCN2A) or other PM proteins. i3Neurons were synchronously infected (MOI 5) with WT or ΔpUL56 HSV-1, or mock infected, and at 24 hpi cells were surface labeled with biotin before the biotinylated proteins were enriched via streptavidin capture and analysed by MS (Fig. 2A). Filtering for proteins with GO cellular compartment annotations “PM,” “cell surface,” or “extracellular region” excluded 716 proteins to result in 1451 PM–enriched host proteins quantified (fig. S7A), with data clustering by infection status rather than biological replicates (fig. S7B). Infection with WT HSV-1 dramatically altered the PM-enriched proteome of i3Neurons, with 138 and 270 cell surface proteins significantly up- or down-regulated, respectively (Fig. 2B, fig. S8, and data S4). Strikingly, 28 proteins were less than a quarter as abundant in the surface-enriched fraction of infected versus mock-infected neurons. These included voltage-gated sodium, potassium, and calcium channel proteins, as well as proteins involved in cell adhesion and receptor transduction, plus the known target of HSV-1 surface down-regulation nectin-1 (*47*). The voltage-gated sodium channel Na_V_1.2 (SCN2A), a target of pUL56-dependent degradation in the whole-cell proteomics, was one of the most highly down-regulated proteins in the surface-enriched fraction, suggesting that pUL56 mediates its PM removal and subsequent degradation. Comparison between WT and ΔpUL56 HSV-1 infections confirmed that pUL56 mediates the down-regulation of Na_V_1.2 (SCN2A) and 13 other voltage-gated ion channel proteins, plus the ionotropic glutamate receptor GRIA2 (Fig. 2C). Thirty-one proteins were more than twofold up-regulated in the PM-enriched fraction of WT HSV-1 versus mock-infected cells, including 7 where the abundance declined back to mock levels in neurons infected with ΔpUL56 HSV-1. Of these seven, two belong to the ANKRD13 family of proteins that mediate the endocytic removal of ubiquitinated PM proteins from the cell surface (*48*). Examples of proteins down-regulated in the surface-enriched fraction during infection with both WT and ΔpUL56 HSV-1 (nectin-1), that were down-regulated by WT HSV-1 but rescued in ΔpUL56 infection (ion channel proteins), or that were up-regulated only during WT HSV-1 infection (ANKRD13 family proteins) are shown in Fig. 2D, and the full PM-enriched proteomics dataset can be interrogated using an interactive plotter worksheet in data S1. GO analysis of the proteins significantly up-regulated in either the WT-versus-mock or ΔpUL56-versus-WT HSV-1 infections did not reveal any significantly enriched molecular function terms. However, among significantly down-regulated proteins, many terms were enriched (Fig. 2, E and F), including multiple terms related to voltage-gated ion channel activity. Previous studies have reported down-regulation of specific ion channels and/or disrupted electrical activity following HSV-1 infection of sensory or CNs (*49*–*57*). However, the full extent of ion channel modulation and the underlying mechanism was unclear.

**Fig. 2.**
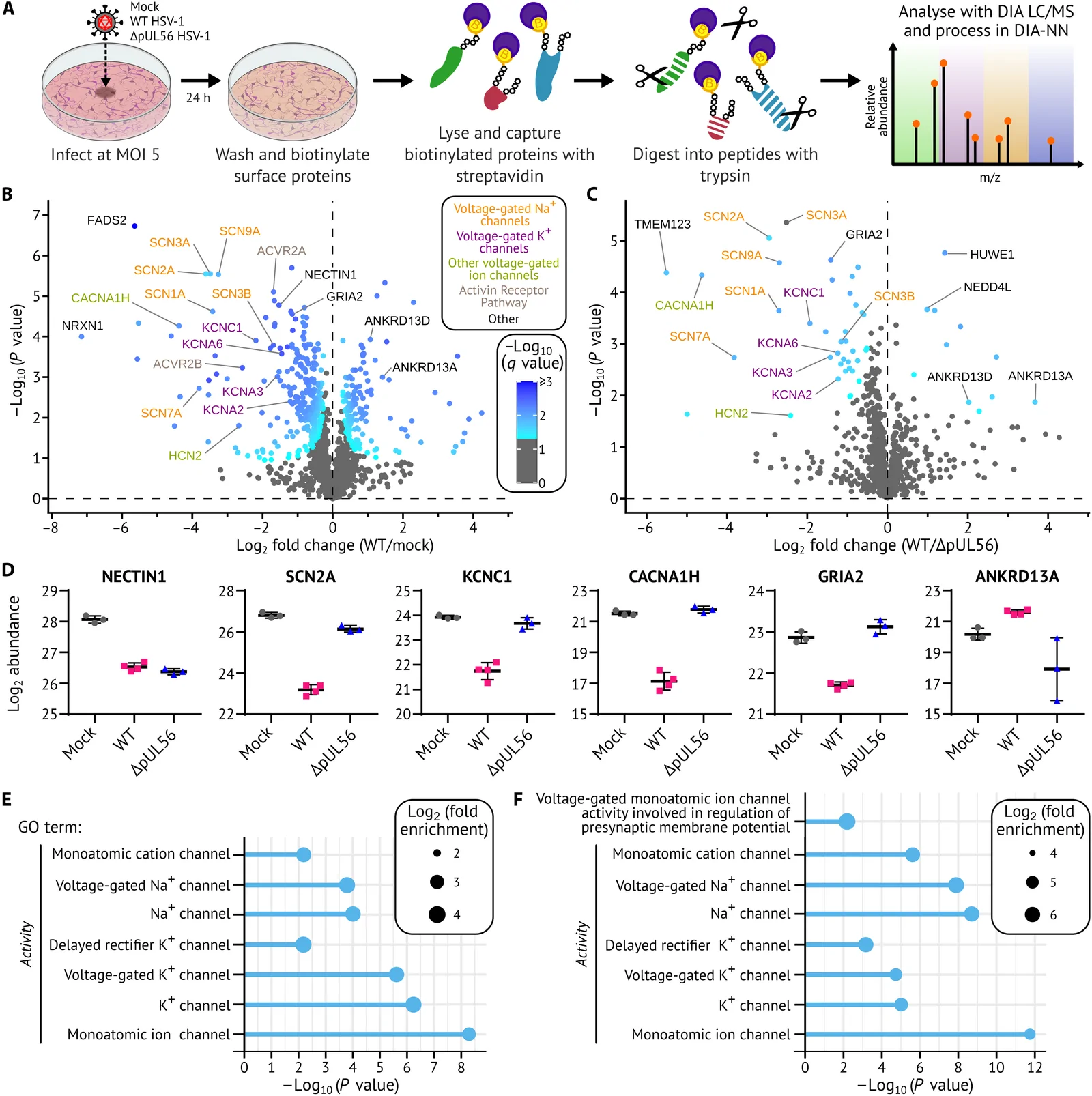
pUL56 reduces the PM abundance of voltage-gated ion channels. (**A**) Schematic of the PM proteomics workflow using day 14 i3Neurons. (**B** and **C**) Change in PM-enriched protein abundance at 24 hpi, showing (B) WT versus mock-infection, or (C) WT versus ΔpUL56 HSV-1 infection. Horizontal axis shows average fold change and vertical axis shows significance (two-sided *t* test) for three (mock and ΔpUL56) or four (WT) independent experiments. Proteins are colored by FDR-corrected significance and selected proteins with significantly altered abundance are labeled. (**D**) PM-enriched protein abundance of HSV-1 entry receptor nectin-1 (NECTIN1), voltage-gated Na^+^ (SCN2A), K^+^ (KCNC1), and Ca^2+^ (CACNA1H) ion channels, GRIA2, and ubiquitin reader protein ANKRD13A in mock-infected versus WT or ΔpUL56 HSV-1–infected i3Neurons. Individual data points and mean abundance ± SD are shown. (**E** and **F**) Enrichment of GO molecular function terms among proteins significantly down-regulated from the PM in (E) WT versus mock-infection, or (F) WT versus ΔpUL56 HSV-1 infection. Bubble sizes represent magnitude of enrichment and significance (FDR-adjusted *P* value) is plotted on the horizontal axis.

To assess whether pUL56 stimulates degradation of ion channels, i3Neurons were synchronously infected (MOI 5) with WT or ΔpUL56 HSV-1, or mock infected. Cells were treated at 6 hpi with the V-ATPase inhibitor bafilomycin A1 (to inhibit lysosomal protein degradation), the proteasomal inhibitor MG132, or carrier [dimethyl sulfoxide (DMSO)], and lysates were analyzed by quantitative MS at 24 hpi (Fig. 3A). In total, 6721 proteins were identified in all samples analyzed across two biological replicates (fig. S9 and data S5), and the full proteolysis inhibition dataset can be interrogated using an interactive plotter worksheet in data S1. Expression of HSV-1 VP5 and VP13/14 was unchanged in the presence of MG132 and slightly reduced in the presence of bafilomycin A1 (Fig. 3B), confirming that neither treatment prevents HSV-1 late gene expression. Treatment of WT HSV-1–infected cells with bafilomycin A1 and MG132 rescues the abundance of voltage-gated ion channels Na_V_1.2 (SCN2A), Na_V_1.3 (SCN3A), Na_V_1.7 (SCN9A), and HCN2 to levels observed during ΔpUL56 HSV-1 infection, confirming that pUL56 stimulates their degradation (Fig. 3B). In contrast, the abundance of glutamate receptor 2 (GRIA2) is unchanged, suggesting that its reduced abundance in the PM-enriched fraction is a result of altered intracellular trafficking rather than enhanced proteolysis. Treatment with proteolysis inhibitors also confirmed the pUL56-dependent degradation of cytoplasmic factors GOPC and PLCL2 in infected cells plus HSV-1–dependent degradation of STMN2, PRKDC, and KIF1A (Fig. 3B). The significant changes in volage-gated ion channel abundance between WT and ΔpUL56 HSV-1–infected neurons, and the pUL56-dependent rescue of voltage-gated ion channels in the presence of proteolysis inhibitors, strongly suggested that pUL56 disrupts neuronal electrical activity by degrading voltage-gated ion channels and thereby altering ion flux.

**Fig. 3.**
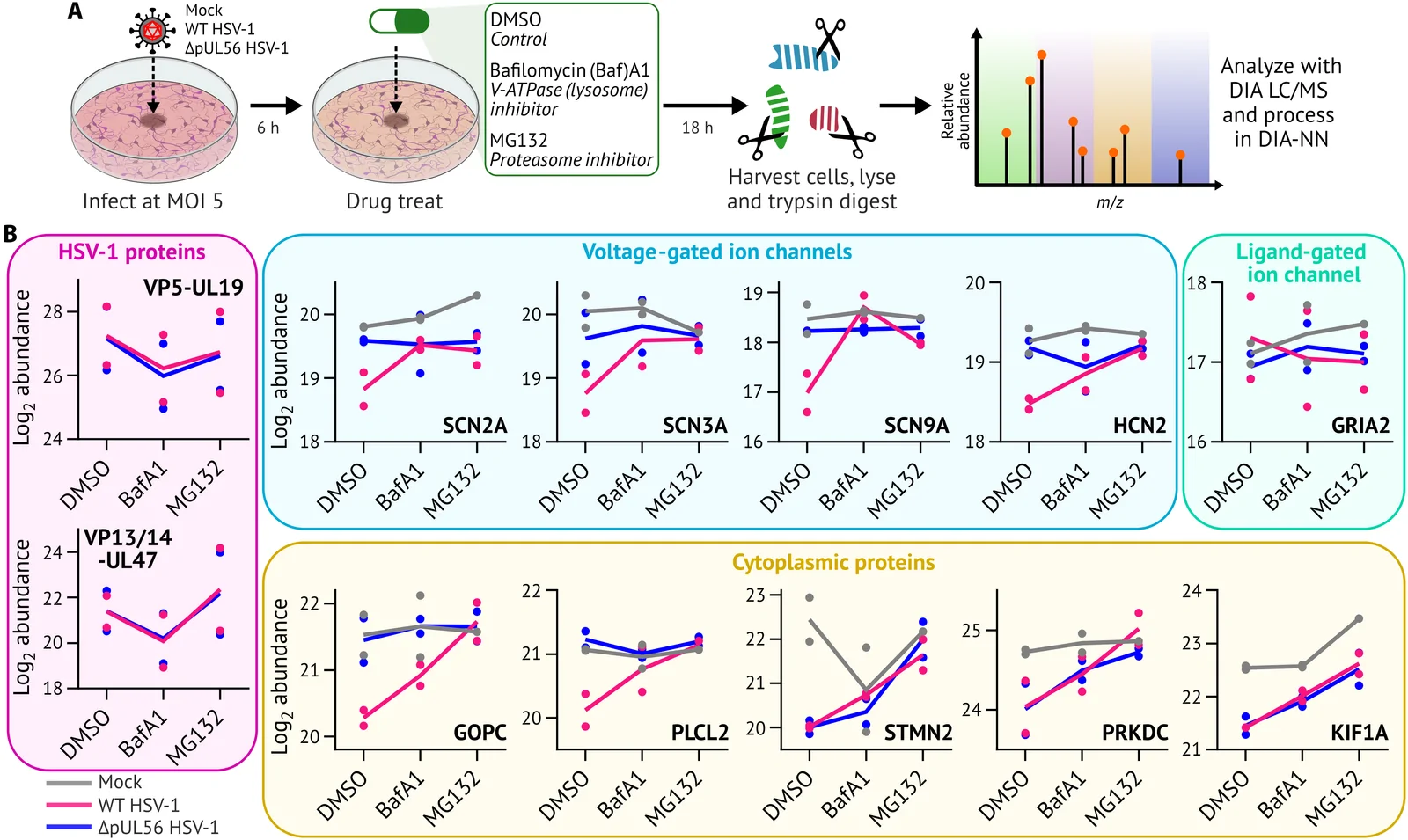
pUL56 stimulates proteolysis of voltage-gated ion channels. (**A**) Schematic of the whole-cell proteomics workflow using day 14 i3Neurons in the presence of proteolysis inhibitors bafilomycin A1 (BafA1), a V-ATPase inhibitor that prevents lysosomal acidification and thus lysosomal protease activity, the proteasome inhibitor MG132, or carrier (DMSO). (**B**) Abundances of selected voltage- and ligand-gated ion channels, HSV-1 late gene products, and cytoplasmic cellular proteins following infection with WT or ΔpUL56 HSV-1, or mock infection, in the presence of proteolysis inhibitors are shown as individual data points from two independent experiments (one for MG132-treated mock infection).

### pUL56 silences calcium signaling during infection via an interaction with E3 ubiquitin ligases

Like many neurotropic viruses, HSV-1 infection alters neuronal electrophysiology (*49*, *50*, *58*–*63*) but the underlying mechanism was unknown. To investigate the consequence of pUL56 altering the abundance of voltage-gated ion channels at the neuronal surface, changes to calcium signaling during infection were analyzed as a proxy for neuronal electrical activity (*64*). i3Neurons are electrically mature after approximately 30 days (*30*), so i3Neurons that had been transduced with a fluorescent calcium biosensor were synchronously infected (MOI 5) on day 42 with WT or ΔpUL56 HSV-1, or mock infected. We hypothesized that pUL56-directed ubiquitination via NEDD4-family E3 ligases (*17*) caused the observed proteolysis of voltage-gated ion channels and that E3 ligase binding would thus be required for HSV-1–mediated changes in electrical activity. Therefore, neurons were also infected with pUL56 AAXA123 HSV-1 where all three PPXY motifs had been mutated to reduce E3 ligase binding affinity (*17*).

Preliminary experiments showed that repeated calcium-flux kinetic measurements require de novo protein expression to replace biosensor molecules that bleach during the fluorescent imaging and that by 24 hpi with HSV-1 this fluorescence recovery was inhibited, presumably by host shutoff. Therefore, calcium imaging was conducted at 12, 24, and 48 hpi on separate wells of i3Neurons (Fig. 4A). At 50 hpi, the wells measured at 12 hpi were treated for 1 hour with 1 μM tetrodotoxin (TTX) to eliminate electrical activity via inhibiting sodium channels before being reimaged. Immunostaining of the wells measured at 24 hpi confirmed near-100% infection (fig. S10). Calcium ion flux remained unaffected by HSV-1 infection at 12 hpi (Fig. 4, B and C, and movie S1). However, by 24 hpi, synchronous firing was entirely abolished in cells infected with WT HSV-1 while it persisted at levels comparable to mock-infected samples in neurons infected with either ΔpUL56 or pUL56 AAXA123 HSV-1 (Fig. 4B and movie S2). The number of active neurons declined in all infections, but WT HSV-1 caused a substantially greater reduction at 24 hpi and near-complete cessation of neuronal activity by 48 hpi (Fig. 4C and movie S3). In contrast, approximately two-thirds of the cells infected with ΔpUL56 or pUL56 AAXA123 HSV-1 remained active at 48 hpi. Representative traces of calcium biosensor intensity at 48 hpi illustrate how mock-infected neurons and those infected with ΔpUL56 or pUL56 AAXA123 HSV-1 exhibit synchronous intracellular calcium bursts, albeit with a reduced peak amplitude in the infected neurons, whereas neurons infected with WT HSV-1 had no synchronous activity, akin to treatment of neurons with TTX (Fig. 4D). pUL56 is thus required for the dramatic inhibition of neuronal calcium signaling in infected neurons, and this inhibition requires the PPXY motifs of pUL56 that mediate association with ubiquitin E3 ligases.

**Fig. 4.**
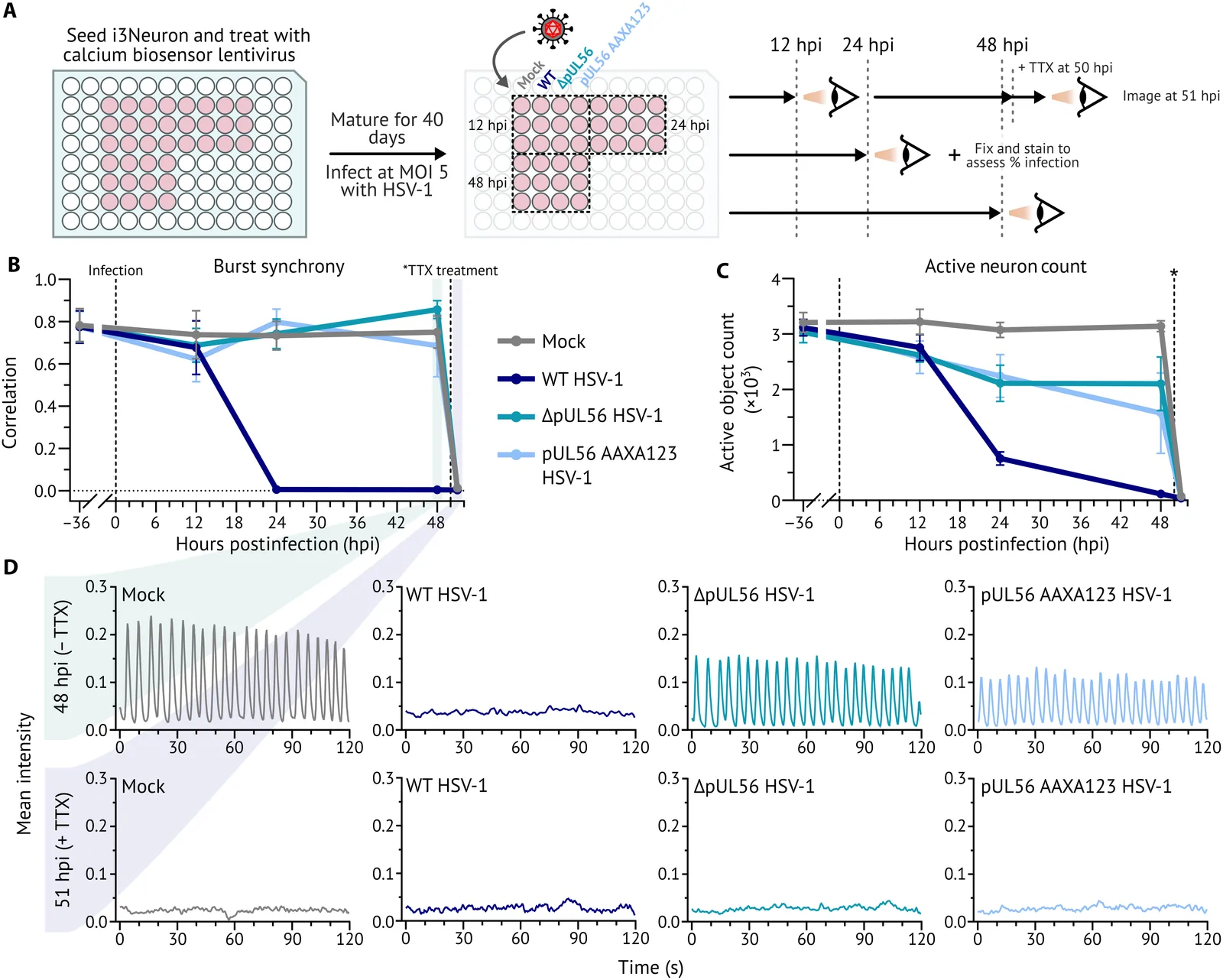
HSV-1 infection disrupts calcium signaling in i3Neurons. (**A**) Schematic diagram of the experimental design. Calcium biosensor transduced day 40 i3Neurons were infected with WT, ΔpUL56, or pUL56 AAXA123 HSV-1 (MOI 5), or mock-infected, and imaged at 12, 24, or 48 hpi. Cells imaged at 12 hpi were treated with 1 μM TTX at 50 hpi and imaged 1 hour later. (**B**) WT HSV-1 infection causes a loss of synchronous calcium bursts by 24 hpi, equivalent to TTX treatment, whereas burst synchrony is not reduced in ΔpUL56 and pUL56 AAXA123 infected cells up to 48 hpi. Means ± SD of three independent experiments performed in technical triplicate are shown. (**C**) The number of actively signaling neurons steadily decreases following ΔpUL56 and pUL56 AAXA123 HSV-1 infection, whereas it drops dramatically at 24 hpi with WT HSV-1. (**D**) Representative plots of mean calcium biosensor signal versus time, showing complete loss of calcium signaling at 48 hpi with WT HSV-1, or at 51 hpi following 1 hour of treatment of i3Neurons with TTX, but not in cells infected with ΔpUL56 or pUL56 AAXA123 HSV-1 at 48 hpi.

### pUL56 alone is sufficient to abolish neuronal electrical activity

Altered neuronal activity during HSV-1 infection could result from direct pUL56-stimulated degradation of voltage-gated ion channels, from mistrafficking of these channels as a consequence of pUL56-stimulated GOPC degradation, or via pUL56 directing the activity of a different viral effector protein. To differentiate between these possibilities, i3Neuron cell lines were generated where either GOPC expression was knocked down or pUL56 alone was expressed. Catalytically dead Cas9 (dCas9) integrated in the i3Neuron iPSCs was exploited for CRISPR interference (CRISPRi)–mediated GOPC knockdown, via transduction of two distinct CRISPRi guides targeting early exons of GOPC to generate two distinct cell lines (GOPC KD1 and KD2). An established i3Neuron cell line containing a scrambled (SCRM) nontargeting guide line was used as a control (*30*). Stable genomic integration of expression cassettes for pUL56 or pUL56 AAXA123, both with N-terminal hemagglutinin (HA) epitope tags to aid detection, was achieved via cotransfection using a piggyBac transposase (*65*, *66*). Quantitative polymerase chain reaction (qPCR) of i3Neurons at day 42 confirmed successful GOPC knockdown (Fig. 5A), and immunoblot analysis confirmed both the absence of GOPC and the expression of pUL56 (Fig. 5B) at higher levels than observed during HSV-1 infection (fig. S11). As expected, the abundance of GOPC was reduced in i3Neurons expressing HA-pUL56 but not in cells expressing HA-pUL56 AAXA123 (*17*).

**Fig. 5.**
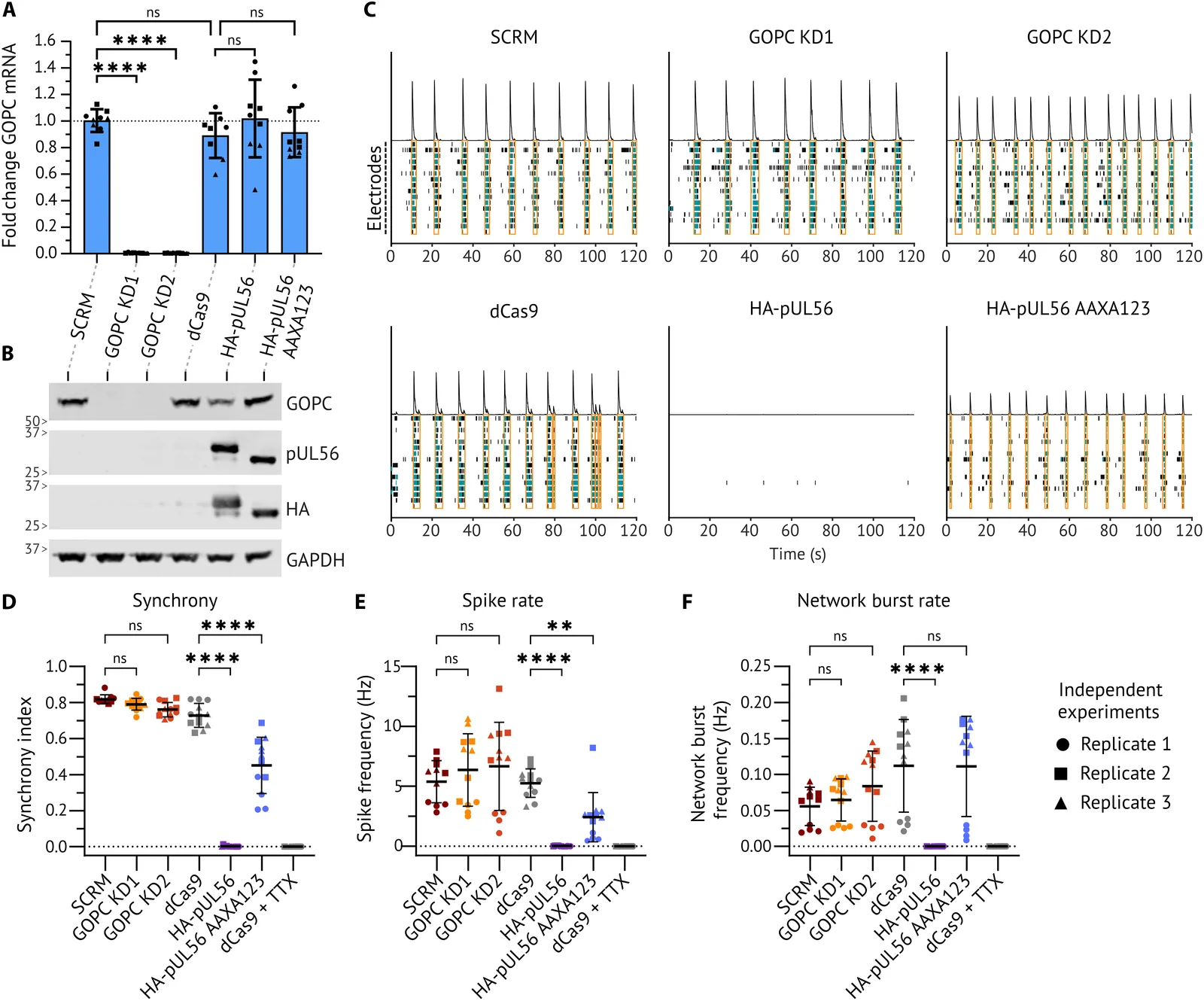
pUL56 halts electrical signaling through a GOPC-independent mechanism. (**A** and **B**) Validation of day 42 i3Neurons expressing catalytically dead (d)Cas9 that have been stably transduced with guides targeting GOPC (GOPC KD1 and KD2), or a nontargeting control (SCRM), or that stably express HA-tagged pUL56 or pUL56 AAXA123. (A) Fold change in GOPC expression, as assessed by qPCR. Means ± SD of three independent experiments, each in technical triplicate, are shown as circles, squares, and triangles. ns, no significance; ****P < 0.0001; one-way analysis of variance (ANOVA) with Sidak’s multiple comparisons test. (B) Immunoblot showing pUL56 expression in HA-pUL56 and HA-pUL56 AAXA123 i3Neurons, and loss of GOPC signal in GOPC KD1 and KD2. Compared to parental cells (dCas9), GOPC abundance is reduced in HA-pUL56 but not HA-pUL56 AAXA123 i3Neurons. Images are representative of one (SCRM and GOPC KDs) or two [dCas9 and HA-pUL56 (AAXA123)] independent experiments. (**C**) Representative MEA raster plots of i3Neuron electrical activity at day 42. For each cell line, frequency histograms of electrical spikes are shown (line trace, top), along with individual spikes (black) and bursts (>5 spikes within 100 ms, cyan) for each electrode (below). Network bursts, defined as simultaneous bursts on >35% of electrodes in the well, are boxed in orange. (**D** to **F**) Quantitation of (D) spike synchrony, (E) spike rate, and (F) network burst rate from MEA recordings at day 42. HA-pUL56 expression severely impairs electrical activity, which is partially rescued in the HA-pUL56 AAXA123 line. For the parental (dCas9) line, activity was recorded before and after 1 hour of incubation with 1 μM TTX. Means ± SD and individual data points from three biological replicates, each performed in technical quadruplicate, are shown as circles, squares, and triangles. ns, no significance; **P < 0.0001; ****P < 0.0001; paired measurements one-way ANOVA with Sidak’s multiple comparisons test.

The electrical activity of GOPC knockdown and pUL56-expressing i3Neurons was examined using multielectrode arrays (MEAs). Electrical activity was recorded on day 42 and is shown in Fig. 5C as raster plots for each cell line, illustrating electrical activity as spikes (black line), bursts (cyan, >5 spikes measured on an electrode within 100 ms), and network bursts (orange box, bursts detected on >35% of electrodes), plus a spike frequency histogram. Electrically healthy i3Neurons evolve regular, high-frequency spike trains that produce numerous bursts and network bursts (Fig. 5C, SCRM and dCas9). Neither of the GOPC knockdown cell lines had reduced electrical activity compared to the SCRM control, whereas the expression of HA-pUL56 almost completely abolished spike activity. The HA-pUL56 AAXA123 cell line had an intermediate phenotype, with electrical activity decreased but still evident. The firing synchrony (Fig. 5D), spike rate (Fig. 5E), and network burst rate (Fig. 5F) of each cell line were quantified, alongside recordings of the dCas9 neurons following treatment with TTX. There was an absence of synchronous firing in i3Neurons expressing HA-pUL56, similar to cells treated with TTX, whereas synchrony was partially restored in cells expressing HA-pUL56 AAXA123. The spike rate was similarly affected, with no activity observed in HA-pUL56 i3Neurons but some rescue of activity in HA-pUL56 AAXA123 i3Neurons. The network burst frequency of HA-pUL56 AAXA123 i3Neurons was similar to the parental (dCas9) cells, as opposed to HA-pUL56 i3Neurons where no network bursts were observed. Collectively, these data demonstrate that pUL56 expression is sufficient to abolish electrical activity in neurons, and the partial rescue observed in the HA-pUL56 AAXA123 expressing cells confirms that neuronal silencing depends on the ability of pUL56 to efficiently bind ubiquitin E3 ligases. Relative to SCRM i3Neurons, neither GOPC KD1 nor KD2 significantly altered neuronal electrical activity, implying that GOPC does not contribute to the removal of voltage-gated ion channels from the PM.

## DISCUSSION

This study identifies pUL56 as a major effector of PM remodeling during HSV-1 infection of human CNs. We demonstrate that pUL56 mediates the removal of voltage-gated ion channels from the PM-enriched fraction of neurons, thereby suppressing electrical signaling during infection. Inhibition of proteolysis restores the abundance of several such ion channels, consistent with their specific targeting for degradation. Infection-induced inhibition of calcium ion flux is reversed when the PPXY E3 ligase–binding motifs of pUL56 are mutated, and pUL56 does not require any other viral proteins to abolish neuronal electrical function. Our findings therefore establish that pUL56 is both necessary and sufficient to silence the electrical activity of neurons.

While HSV-1 induced substantial proteomic and transcriptomic changes in infected neurons, it did not elicit an interferon-based antiviral response as there was no coordinated up-regulation of antiviral genes. This is consistent with our previous observation that HSV-1 infection of i3Neurons is not self-limiting (*27*). However, there was a rapid decrease in the abundance of several proteins implicated in microtubule assembly and trafficking, including multiple stathmin family members and the kinesin-3 family protein KIF1A (*35*, *67*–*69*). These effects are not caused by cell death at 30 hpi, as i3Neurons persist for over 1 week following infection with HSV-1 (*27*) and cells infected with ΔpUL56 or pUL56 AAXA123 HSV-1 retain synchronous calcium ion flux for at least 48 hpi (Fig. 4). Our results align with similar experiments performed in cerebral organoids, where STMN2 was also heavily down-regulated at both the protein and transcription level following HSV-1 infection (*23*), but we show that degradation of the stathmin-family proteins also occurred in response to SFV4 infection (Fig. 1E). These changes could thus represent a general but concerted antiviral or injury response. Inhibiting axonal transport would directly affect virus particle movement and it would potentiate programmed axonal degradation (*70*), both acting to impair virus spread. Low STMN2 abundance in motor neurons caused by loss of TDP-43 nuclear localization is a hallmark of amyotrophic lateral sclerosis (ALS), and the loss or depletion of STMN2 is sufficient to drive ALS-like pathology in mice (*71*–*73*). A recent preprint confirms that HSV-1 seropositivity is significantly associated with increased ALS risk (*74*). While this study implicates the viral E3 ligase ICP0 in driving TDP-43 mislocalization and aggregation, STMN2 abundance was not investigated. Whether STMN2 degradation is a consequence of TDP-43 mislocalization or represents an independent driver of ALS in the context of HSV-1 infection remains to be determined, although the rescue in the presence of MG132 (Fig. 3B) suggests a direct role for virus-induced proteolysis in reducing STMN2 abundance.

The observed down-regulation of KIF1A was specific to HSV-1 infection. While KIF1A has previously been shown to bind HSV-2 pUL56 (*75*), the degradation we observe is not induced by HSV-1 pUL56 since KIF1A was lost at an equivalent rate in WT or ΔpUL56 infected neurons (Fig. 1I) and is rescued similarly when WT or ΔpUL56-infected cells are treated with MG132 (Fig. 3B). KIF1A is a subunit of the kinesin-3 motor protein that mediates anterograde transport of cargo in neurons (*69*), including nascent particles of HSV-1 (*76*) and the related alphaherpesvirus pseudorabies virus (*77*). A direct interaction of KIF1A with pseudorabies virus pUS9 stimulates both virion axonal transport and KIF1A degradation (*77*), with this degradation suggested to occur at synaptic termini after cargo delivery (*78*, *79*). The increase in KIF1A abundance observed when mock-infected i3Neurons are treated with MG132 (Fig. 3B) is consistent with cargo delivery stimulating its degradation. pUS9 is not expressed in HSV-1 strain KOS used in our study (*80*), but pUS9 is not absolutely required for HSV-1 axonal transport (*81*). The HSV-1–specific KIF1A degradation we observe is thus likely to be a result of degradation following synaptic delivery of nascent virions and other cargo, plus lowered KIF1A mRNA abundance following virus infection (Fig. 1H).

For many genes, the transcript and protein abundance changes observed during infection differ (Fig. 1G), highlighting extensive posttranscriptional changes during HSV-1 infection. Of the 28 proteins with significantly altered abundance identified in a previous proteomic study of HSV-1 infection using mixed mouse primary cortical cells (*82*), the only common change we observed was significant down-regulation of the Kelch-like ubiquitin E3 ligase KLHL9. Ubiquitin ligase–binding functions are enriched among proteins significantly down-regulated at 30 hpi (Fig. 1F), and inhibition of proteolysis restores the abundance of many proteins at 24 hpi (Fig. 3B and fig. S9), consistent with previous reports of HSV-1 hijacking host ubiquitin pathways to eliminate antiviral factors and reshape the infected cell environment (*17*). pUL56 has previously been identified as an adaptor for the ubiquitin-proteasome axis, binding to E3 ubiquitin ligases to target proteins such as GOPC for degradation (*17*). The loss of ion channels from the PM-enriched fraction was rescued in ΔpUL56 infection, and synchronous calcium ion flux was restored either when pUL56 was absent during infection or when its PPXY motifs were mutated. The loss of synchrony mimics TTX treatment, strongly indicating that suppression of neuronal activity arises from pUL56-directed loss of cell surface voltage–gated ion channel activity.

We demonstrate that pUL56 expression alone is sufficient to abolish neuronal electrical activity. MEA recordings show that pUL56, in the absence of any other viral gene, eliminates spikes, bursts, and network-level synchrony, while mutation of the pUL56 PPXY motifs produces a partial rescue. In contrast, GOPC knockdown has no impact on electrical activity. This is similar to the observation in human embryonic kidney (HEK) 293T cells that pUL56 mediates degradation of volume-regulated anion channels in an E3 ligase–dependent but GOPC-independent manner (*83*). In i3Neurons, we propose that pUL56 colocalizes with voltage-gated ion channels to cause their ubiquitination and subsequent removal from the PM. The observation that the whole-cell abundance of some voltage-gated ion channels is reduced in HSV-1–infected cells only when pUL56 is present, and that their abundance is restored upon treatment with proteolysis inhibitors, supports this model. The PM localization of most of the targeted ion channels is controlled by ubiquitination by NEDD4-family E3 ligases (*84*–*89*), the same family that pUL56 binds (*17*, *90*–*92*). Furthermore, the E3 ligases NEDD4L and HUWE1 are more abundant in the cell surface–enriched proteome during WT infection, suggesting their recruitment by pUL56. Two members of the ANKRD13 family of ubiquitin-reader proteins were also up-regulated in the PM-enriched fraction during WT infection, consistent with their recruitment either by pUL56 directly or by targets of pUL56-directed ubiquitination. These proteins form complexes that specifically bind to lysine-63-linked polyubiquitin, a form preferred by NEDD4 ligases (*93*, *94*), on surface proteins, causing their endocytic internalization and lysosomal degradation (*48*). This evidence supports a model in which pUL56 recruits E3 ligases to the PM to ubiquitinate voltage-gated ion channels, likely via K63 polyubiquitination, and then recruits ANKRD13 proteins to mediate their removal from the neuronal surface, although this remains to be shown directly. In this context, the MG132-mediated proteasomal inhibition would also reduce ubiquitin recycling, depleting the free cellular ubiquitin pool (*95*), and could explain the observed rescue of voltage-gated ion channel abundance. We note that some ion channels like GRIA2 are not degraded in HSV-1–infected cells, suggesting that their PM down-regulation may be caused by altered trafficking rather than stimulated degradation. Further work is therefore required to define the precise mechanism(s) via which pUL56 modulates the trafficking and/or degradation of ion channels to cause neuronal silencing.

The vast majority of adults worldwide are infected with HSV-1 (*1*). Although it remains unclear whether the virus can establish latent infections in human CNs, there is extensive evidence of HSV-1 DNA in the brain of individuals that have not experienced clinical HSE (*96*, *97*). These observations are consistent with sporadic subclinical infections of the CNS that arise following reactivation of latent virus in the trigeminal ganglia and are either restricted by intrinsic immune factors (*98*) or controlled via innate immune sensing (*99*, *100*). Our data suggest that during (subclinical) lytic infection, the affected neurons will be electrically silenced within 24 hours. Although it remains unclear whether disruption of neuronal firing is advantageous for virus replication and spread, disruption of electrical activity is clearly detrimental for brain function and represents a major contributing factor to many neurological and degenerative disorders, including AD (*101*–*104*). A recent study demonstrated that subclinical neonatal infection with HSV-1 leads to persistent CNS infection and poor cognitive performance of adult mice (*105*). Epidemiological studies have linked HSV-1 seropositivity to a higher risk of dementia (*106*–*108*) and suggested that HSV antiviral treatment may reduce dementia risk (*109*), although not all studies confirm this association (*110*). Treatment with the acyclovir prodrug valacyclovir improved memory and cognitive function in HSV-seropositive patients with schizophrenia (*111*), but a recent randomized controlled trial demonstrated no effect of valacyclovir on cognitive worsening of HSV-seropositive patients with early symptomatic AD (*112*). While neuronal silencing by pUL56 during subclinical CNS infections provides a compelling molecular mechanism that could link HSV-1 infection with cognitive impairment, the extent to which HSV-1 infection (and thus pUL56 expression) predisposes individuals to AD or alters its preclinical progression remains to be confirmed.

Our study defines pUL56 as a potent viral suppressor of neuronal excitability, acting through ubiquitin-dependent removal of voltage-gated ion channels from the neuronal surface. The discovery that a single viral protein can shut down neuronal networks provides insight into how neurotropic viruses manipulate the nervous system. There is increasing interest in the use of nonreplicative (nr)HSV-1 as a neurotropic gene delivery vector (*113*). While modern nrHSV-1 vectors show very low levels of viral gene expression (*114*), there have been reports of virion-delivered proteins altering neuronal activity (*115*). Given that pUL56 is packaged into HSV-1 virions (*116*), the safety of nrHSV-1 vectors might be further enhanced by removal of the UL56 gene.

## MATERIALS AND METHODS

### Cell culture

All cells were incubated in a humidified 5% CO_2_ atmosphere at 37°C and regularly tested to confirm the absence of mycoplasma. iPSCs and i3Neurons (RRID:CVCL_C7XJ) were maintained and differentiated as detailed in (*27*). Briefly, iPSCs containing a doxycycline-inducible Ngn2 transcription factor (*25*) were maintained in Essential 8 medium (Gibco) on Matrigel (Corning)–coated plates. Differentiation was initiated by addition of doxycycline (2 μg/ml) in Dulbecco’s modified Eagle’s medium (DMEM)/F-12 (Gibco) supplemented with 1× N2 (Gibco), 1× nonessential amino acids (Gibco), and 2 mM l-glutamine (Gibco). For the first day of differentiation, the medium was supplemented with 50 nM Chroman 1 (Bio-Techne), and the medium was changed daily. On day 3, the cells were dissociated with StemPro Accutase (Gibco) and frozen at −70°C in 90% v/v KnockOut Serum Replacement (Gibco) plus 10% v/v DMSO. Thawed day 3 i3Neuron precursors were matured in CN medium, comprising Neurobasal Plus Medium (Gibco) supplemented with 1× B27 (Gibco), brain-derived neurotrophic factor (10 ng/ml, PeproTech), Neurotrophin-3 (10 ng/ml, PeproTech), and laminin (1 μg/ml, Gibco), on surfaces precoated with poly-l-ornithine (100 μg/ml, PLO). CN medium was supplemented with doxycycline (2 μg/ml) only when seeding the cells. Half media changes were performed twice weekly until day 14 and then thrice weekly. Vero (RRID:CVCL_0574) and HEK293T (RRID:CVCL_0063) cells were grown in DMEM with 2 mM l-glutamine and 10% v/v fetal bovine serum (complete DMEM). BHK21 (RRID:CVCL_1914) cells were grown in DMEM with 2 mM l-glutamine, 5% v/v fetal bovine serum, 0.3% tryptose phosphate broth, penicillin (100 U/ml), and streptomycin (100 μg/ml).

### GOPC CRISPRi knockdown

CRISPRi knockdown cell lines were generated via lentivirus transduction. Guide RNAs targeting GOPC were designed using the CRISPick server (Broad Institute) to target exon1. Primers encoding the guides (table S1) with complementary overhangs were cloned into the BpiI restriction site of the plasmid pKLV. Lentiviruses were generated, and iPSCs were transduced as described in (*30*). After 1 week of puromycin selection, the GOPC knockdown i3Neurons were differentiated as described above.

### pUL56 stable transfection

N-terminally HA-tagged pUL56 was amplified by PCR (table S1) and cloned via NEBuilder HiFi DNA assembly (New England Biolabs) into vector iC10, a gift from M. Nelson and M. Ward (National Institutes of Health). HA-tagged pUL56-AAXA (*17*) was subcloned into this vector following restriction digest using NotI and XhoI sites introduced when cloning HA-pUL56. iPSCs were cotransfected with a 1:3 ratio of the K13 piggyBac transposase plasmid, a gift from M. Nelson and M. Ward (National Institutes of Health), and the relevant expression plasmid using Lipofectamine Stem (Invitrogen). Two days posttransfection, hygromycin B (125 μg/ml) was added to the E8 medium and selection was maintained for 1 week before surviving cells were differentiated as described above.

### Viruses

Stocks of WT, ΔpUL56, and pUL56 AAXA HSV-1 strain KOS that had been reconstituted from bacterial artificial chromosomes (*17*) were grown on Vero cells, as were HSV-1 strains 17 and SC16. HA-pUL56 and HA-pUL56 AAXA123 HSV-1 were generated by two-step red recombination (*117*) of pUL56 and pUL56 AAXA123 viruses, respectively, using the primers shown in table S1. All HSV-1 stocks were filter-purified from the supernatant of Vero cells treated with NaCl and dextran sulfate as described in (*27*). SFV4 was rescued from the plasmid pSP6-SFV4 (*118*) in BHK21 cells and further amplified using MOI 0.01 infection. Media-derived virus was clarified using 0.4-μm filter and concentrated using Amicon Ultra centrifugal filters with 100-kDa molecular weight cutoff. The resulting virus was titrated on BHK21 by plaque assay (*119*).

### Single-step growth curves

Day 14 i3Neurons grown in 24-well plates were infected (MOI 5) for 1 hour with WT or ΔpUL56 HSV-1, incubated for 1 min with citric acid, and washed three times with phosphate-buffered saline (PBS) before being overlaid with conditioned medium as described in (*27*). At 3, 6, 9, 12, 18, 30, and 48 hpi, neurons were harvested by freezing-thawing and virus titers were assessed by plaque assay on Vero cells (*27*).

### Immunoblotting

Day 14 i3Neurons were infected (MOI 10) for 1 hour with HSV-1 strains KOS, S17, and SC16, or SFV4 as described in (*27*). At 16 hpi, cell lysates were prepared by detaching neuronal cell layers with ice-cold PBS containing 1% v/v protease-inhibitor cocktail, centrifugation to collect cells and lysis in radioimmunoprecipitation assay buffer as described in (*27*). Following separation by SDS–polyacrylamide gel electrophoresis, proteins were transferred onto 0.45-μm Protran nitrocellulose membranes (Cytivia) using the Mini-PROTEAN system (Bio-Rad) and blocked in TBS [50 mM tris (pH 7.6) and 150 mM NaCl] supplemented with 5% w/v skim milk powder (Marvel). Primary and secondary antibody incubations were performed in TBS supplemented with 0.1% TWEEN-20 and 5% w/v skim milk powder using the following antibodies: HSV-1 VP5, (*120*); SV4 CP, provided by A. Merits (*119*); KIF1A, BD Transduction Laboratories, 612094; cFOS, Cell Signaling Technologies, 2250T; GOPC, Abcam, Ab133472; STMN2, Novus Biologicals, NBP1-49461; STMN3, ProteinTech, 11311-1-AP; STMN4, ProteinTech, 12027-1-AP; glyceraldehyde-3-phosphate dehydrogenase (GAPDH), GeneTex, GTX28245; HSV-1 pUL56, (*17*); HA, Cell Signalling Technologies, C29F4; anti-rabbit 800, LI-COR, 926-32213; anti-rabbit 680, LI-COR, 926-68023; anti-mouse 800, LI-COR, 926-32210; anti-mouse 680, LI-COR, 926-68020. Immunoblots were imaged using an Odyssey CLx (LI-COR) and analyzed using Image Studio Lite (LI-COR).

### Quantitative PCR

Cells were lysed, RNA extracted using the PureLink RNA Mini extraction kit (Invitrogen), cDNA generated, and qPCR was performed using TaqMan master mix and FAM-labeled assay probes (Bio-Rad) for GAPDH (Hs02786624_g1) and GOPC (Hs01100473_m1) as described in (*27*). Expression changes were measured using the ΔΔCt method (*121*).

### Quantitative temporal proteomics

Day 3 i3Neurons (3 × 10^5^) were seeded in PLO-treated 24-well plates (two wells per condition per time point per replicate, plus one additional well per infection for microscopy) and matured to day 14 before being synchronously infected (MOI 5) with WT or ΔpUL56 HSV-1, or mock-infected. Inoculum was removed after 1 hour, and cells were washed thrice with PBS before being overlaid with conditioned media. At 3, 6, 12, 18, and 30 hpi, medium was removed and cells were washed once with PBS, which was subsequently aspirated and plates frozen at −70°C. For lysis, plates were thawed and cells scraped into 75 μl of lysis buffer [6 M guanidine HCl and 50 mM Hepes (pH 8.5)]. Lysates from duplicate wells were pooled, snap-frozen in liquid nitrogen, and stored at −70°C. At 18 hpi, the additional wells were fixed and stained for ICP4 (ATCC 58S) to assess percentage of infection as described in (*27*). Lysates were thawed, vortexed extensively, sonicated at maximum power for five cycles of 30 s, and clarified twice by centrifugation (13,000*g*, 10 min, 4°C). Supernatant (150 μl) was reduced (5 mM dithiothreitol, 20 min) and then alkylated (14 mM iodoacetamide, 20 min) at room temperature (RT), protected from light, before quenching with excess dithiothreitol. Samples were diluted fourfold with 200 mM Hepes (pH 8.5) before being supplemented with 3 μl of LysC protease (Promega) for 3 hours at RT. The LysC-digested sample was diluted threefold with 200 mM Hepes (pH 8.5) before addition of 1.5 μg of sequencing-grade trypsin (Promega) and incubation overnight at 37°C on a shaking incubator. The reaction was quenched by addition of 5% v/v formic acid and clarified by centrifugation (21,000*g*, 10 min). Digested peptides were desalted (Pierce Peptide Desalting Spin Columns, Thermo Fisher Scientific), eluted in 50% v/v acetonitrile plus 0.1% trifluoroacetic acid, dried (45°C, 1 Torr) and resuspended in 20 μl of 200 mM Hepes (pH 8.5). The concentration of resuspended peptides was analyzed using the Pierce Quantitative Colorimetric Peptide Assay (Thermo Fisher Scientific) as per the manufacturer’s instructions.

Thirty micrograms of peptides per sample was diluted to a total volume of 14 μl with 200 mM Hepes (pH 8.5), supplemented with 100 μg of TMTpro 18-plex reagents (Thermo Fisher Scientific) in anhydrous acetonitrile (Acros Organics), vortexed, and then incubated at RT for 1 hour. The labeling reaction was quenched with 5% v/v hydroxylamine (Sigma-Aldrich) for 15 min at RT, and then formic acid was added to 1% v/v. Sample labeling was as indicated in table S2. All 18 TMT-labeled samples were combined at a 1:1 ratio, desalted, and analyzed by single injection on an Orbitrap Fusion Lumos (Thermo Fisher Scientific) to estimate label contribution. Samples were combined on the basis of observed reporter ion abundances from the single injection to achieve equal label loading, desalted, and fractionated by high-pH reverse-phase chromatography using an Ultimate 3000 ultrahigh-performance chromatography (UHPLC) system (Thermo Fisher Scientific) equipped with a Kinetex Evo C18 (1.7 μm, 2.1 × 150 mm) column (Phenomenex). The mobile phase used was: HPLC-grade water 3:97 v/v (solvent A); 100% v/v liquid chromatography–MS–grade acetonitrile (solvent B) and 200 mM ammonium formate in HPLC-grade water, pH 10 (solvent C). Eluent C was maintained at 10%, while A and B were altered over the fractionation gradient. The elution gradient was 0 to 19% B in 10 min, 19 to 34% B in 14.25 min, and 34 to 50% B in 8.75 min, followed by a 10-min wash of 90% B. Flow was set at 0.20 ml/min for the first 5 min, while peptides, which were reconstituted in 40 μl of eluent C, were loaded onto the column. Thereafter, the flow rate was increased to 0.40 ml/min for the rest of the protocol. A total of 168 fractions were collected every 0.25 min between 10 and 52 min of the fractionation run. The fractions were concatenated into 12 pools, which were dried in a refrigerated vacuum centrifuge.

MS data were acquired using an Orbitrap Fusion Lumos (Thermo Fisher Scientific) equipped with an EASYspray source and coupled to an RSLC3000 nano UPLC (Thermo Fisher Scientific). Peptides were fractionated using a 50-cm C18 PepMap EASYspray column maintained at 40°C with a solvent flow rate of 300 nl/min. A gradient was formed using solvent A (0.1% formic acid) and solvent B (80% v/v acetonitrile, 0.1% formic acid) rising from 3 to 7% solvent B by 7 min, from 7 to 37% solvent B by 180 min followed by a 4 min wash at 95% solvent B. MS acquisition used a MultiNotch MS3-based TMT method using the following settings: MS1, 380 to 1500Th, 120,000 resolution, 2.0 × 10^5^ automatic gain control (AGC) target, 50-ms maximum injection time; MS2, quadrupole isolation at an isolation width of mass/charge ratio (*m/z*) 0.7, collision-induced dissociation fragmentation [normalized collision energy (NCE) 34] with ion-trap scanning in turbo mode with 1.5 × 10^4^ AGC target; MS3, in synchronous precursor selection mode the top 10 MS2 ions were selected for HCD fragmentation (NCE 45) and scanned in the Orbitrap at a resolution of 60,000 with an AGC target of 1.0 × 10^5^ and a maximum accumulation time of 150 ms. Ions were not accumulated for all parallelizable time. The entire MS/MS/MS cycle had a target time of 3 s. Dynamic exclusion was set to ±10 parts per million for 70 s. MS2 fragmentation was triggered on precursors of 5.0 × 10^3^ counts and above.

Raw mass spectra were processed using MaxQuant version 2.5.2.0 (*122*, *123*) searching against a manually curated HSV-1 strain KOS proteome and the human proteome (UP000005640, accessed on 17.04.2024). Reporter ion intensities were corrected using lot-specific isotopic impurity values (table S3). Default options within MaxQuant were used with match between runs analysis, and QTV whole cell lysate data (fractions 1 to 14) from (*17*) were included as a separate parameter group to improve search power. Data filtering and label normalization were performed in R (Software S1). All potential false positives (contaminants, reverse hits, and proteins identified only by site) were removed. Scale factors for each label were calculated by comparing the column sum of all proteins within a label to the median column sums of all labels. Each reporter ion intensity was scaled for normalization and a log_2_ transformation was applied for downstream analysis using Perseus (*124*). Proteins identified in less than 60% of samples were filtered from the dataset and missing values were imputed. Principal components analysis, histograms, profile plots, and volcano plots were generated using default parameters in Perseus and plotted in R using ggplot2 (https://ggplot2.tidyverse.org/). Significance of fold changes between experimental conditions were assessed using significance analysis of microarrays analysis (S0 = 0.1) with a permutation-based false discovery rate (FDR) of 0.05 to calculate FDR-adjusted *P* value (*q* value) (*125*). The Database for Annotation, Visualisation and Integrated Discovery (DAVID) v2025_2 (*126*) was used to perform GO analysis on genes with significantly altered abundance against a background of all human genes identified in the experiment.

### Quantitative temporal transcriptomics

Day 3 i3Neurons (1.75 × 10^6^) were seeded in PLO-treated six-well plates (one well per condition per time point per replicate) and matured to day 14 before being synchronously infected (MOI 5) with WT or ΔpUL56 HSV-1, or mock-infected. Inoculum was removed after 1 hour, and cells were washed thrice with PBS before being overlaid with conditioned media. At 3, 6, 12, 18, and 30 hpi, medium was removed and cells were washed once with PBS, which was subsequently aspirated and plates frozen at −70°C. RNA was extracted using a PureLink RNA Mini extraction kit (Invitrogen), quantified using a Qubit RNA High Sensitivity (HS) Assay Kit (Invitrogen), and prepared for Oxford Nanopore Technology (ONT) sequencing using the cDNA-PCR Sequencing V14 Barcoding kit (Oxford Nanopore) using 500 ng of RNA per sample and a randomized allocation of barcodes. The concentration of the resultant barcoded cDNA was measured using a Qubit dsDNA HS Assay kit (Invitrogen) and its integrity assessed using an Agilent Bioanalyzer 2100 expert High Sensitivity DNA kit (Agilent) before being pooled 1:1 for all labeled samples to a total of 50 fmol per library. Rapid adaptors were added, and the libraries were sequenced using four PromethION flow cells (Oxford Nanopore), with each library split across two flow cells. Sequencing ran for 3 days and base-calling (Super-accurate basecalling v4.3.0, 400 bases per second) was performed in parallel using the Guppy recurrent neural network algorithm (version 7.4.14 + d47971cbb) provided by ONT. Run data were then summarized by PycoQC (*127*), sequence data were combined, and the data from across the technical replicates were merged. A combined reference cDNA transcriptome file was created containing human (Ensembl v113) and HSV-1 cDNAs. Minimap 2 (version 2.24-r1122) was used to align ONT reads to the combined reference cDNA transcriptome and aligned reads. Salmon version 1.6.0 (*128*, *129*) was used to assign reads to transcripts or gene isoforms and produce a read count matrix. Differential gene expression was performed in R (Software S2) using DESeq2 from Bioconductor (*130*). Raw gene-level counts were imported, and genes with low counts across all samples (median count ≤3) filtered out before normalization. The DESeq2 median-of-ratios method was used to calculate size factors, correcting for variations in sequencing depth and RNA composition bias. Variance estimation and dispersion fitting were conducted using an empirical Bayes shrinkage procedure. To stabilize variance across the dynamic range, counts were transformed using a variance stabilising transformation before t-distributed stochastic neighbor embedding analysis. Differential expression analysis was conducted using Wald tests, with resulting *P* values adjusted for multiple testing via the Benjamini-Hochberg FDR procedure. Profile plots, volcano plots, and abundance plots were generated using ggplot2 and GO analysis was performed using (DAVID) v2025_2 (*126*) with a background of all human genes identified in the experiment.

### PM proteomics

Day 3 i3Neurons (1 × 10^7^) were seeded in PLO-treated 10-cm dishes and matured to day 14 before being synchronously infected (MOI 5) with WT or ΔpUL56 HSV-1, or mock-infected. Inoculum was removed after 1 hour, and cells were washed thrice with PBS before being overlaid with conditioned media. At 24 hpi, cells were washed thrice with ice-cold PBS (pH 7.4) and then overlain with 5 ml of ice-cold PBS supplemented with 1 mM sodium periodate (Sigma-Aldrich), 100 μM aminooxy-biotin (Biotium), and 10 mM aniline (Sigma-Aldrich). Samples were incubated for 30 min at 4°C protected from light before biotinylation was quenched by addition of an equal volume of 2 mM glycerol. Cells were gently washed thrice with ice-cold PBS and then scraped into 1 ml of ice-cold lysis buffer [10 mM tris (pH 7.4), 1% v/v Triton X-100, 150 mM NaCl, 5 mM EDTA, and 1% v/v EDTA-free protease inhibitor cocktail] and transferred to low-bind microcentrifuge tubes (Corning). Cells were lysed for 1 hour and lysates clarified by centrifugation (20,000*g*, 10 min, 4°C), snap-frozen in liquid nitrogen, and stored at −70°C until required. Once all samples were ready for enrichment, lysates were thawed, their protein concentrations measured via bicinchoninic acid assay (Pierce), and protein concentrations were normalized via addition of lysis buffer. Neutravidin beads were washed with lysis buffer before being incubated with samples for 3 hour at 4°C. Beads with enriched samples were washed in SnapCap filter columns (Pierce) on a vacuum manifold 20 times using lysis buffer, 20 times using PBS with 0.5% SDS, and then 10 times with urea buffer comprising 6 M urea and 50 mM triethylammonium bicarbonate (TEAB) pH 8.5 (Thermo Fisher Scientific). Columns were removed, capped, and beads incubated with a reduction/alkylation mixture comprising 10 mM tris(2-carboxyethyl)phosphine (Thermo Fisher Scientific) and 20 mM iodoacetamide in urea buffer for 30 min on a shaking platform protected from light. The beads were then washed on the vacuum manifold a further 10 times with urea buffer and then five times with 50 mM TEAB. Beads were transferred to a microcentrifuge tube, supernatant was removed, and beads were resuspended in 50 μl 50 mM TEAB containing 0.5 μg of sequencing-grade trypsin (Promega). Proteins were digested overnight at 37°C in an incubator shaking at 1000 rpm. The next day, beads were pelleted (500*g*, 5 min) and the supernatant reserved before the beads were washed with 40 μl of 50 mM TEAB and pelleted again. The wash supernatant was combined with the reserved supernatant and samples dried in a vacuum centrifuge.

Digested peptides were subjected to LC/MS2 analysis using a Thermo Q Exactive Plus mass spectrometer (ThermoFisher Scientific) equipped with an EASYspray source and coupled to an RSLC3000 nano UPLC (Thermo Fischer Scientific). Peptides were fractionated using a 50-cm C18 PepMap EASYspray column maintained at 40°C with a solvent flow rate of 300 nl/min. A gradient was formed using solvent A (0.1% formic acid) and solvent B (80% v/v acetonitrile, 0.1% formic acid) rising from 3 to 40% solvent B by 90 min followed by a 5-min wash at 95% solvent B. MS spectra were acquired at 35,000 resolution between *m/z* 350 and 1430 with MS/MS spectra acquired in a DIA fashion. Fragmentation spectra were acquired at 17,500 fwhm following HCD activation with a 30-*m/z* isolation window, 1.0 × 10^6^ AGC target and a loop count of 18. DIA-NN version 1.9.2 (*131*) was used to analyze the resultant spectra, which were searched against an in silico library generated using default parameters from a curated HSV-1 strain KOS proteome and the human proteome (UP000005640, accessed on 06.12.2024). Optimal analysis parameters (mass accuracy and MS1 accuracy) were calculated from a short initial processing of three random samples and then applied to all samples with match between runs, as recommended for proteomic spectra captured on a Q Exactive Plus mass spectrometer. Protein abundances were exported as MaxLFQ scores (*132*). DIA-NN data were preprocessed in R by removing common contaminant proteins and applying a log_2_ transformation (Software S3). PM proteomics data were further filtered (Software S4) to only include proteins annotated within biomaRt (*133*) with GO cellular compartment terms PM (GO:0005886), cell surface (GO:0009986), or extracellular region (GO:0005576) before analysis using Perseus (*124*) as above, with the difference that proteins identified in less than three replicates in at least one infection condition were removed before imputation of missing values.

### Whole-cell proteomics following drug treatment

Day 3 i3Neurons (3 × 10^5^ or 2 × 10^6^) were seeded in PLO-treated 24- or 6-well plates, respectively, and matured to day 14 before being synchronously infected (MOI 5) with WT or ΔpUL56 HSV-1, or mock-infected. Inoculum was removed after 1 hour, and cells were washed thrice with PBS before being overlaid with CN media. At 6 hpi, overlay was supplemented with drug-containing CN medium to give final concentrations of 0.1% DMSO (carrier), 10 μM MG132 plus 0.1% DMSO, or 100 nM bafilomycin A1 plus 0.05% DMSO. At 24 hpi, cells were washed thrice with ice-cold PBS and PBS was fully removed before cells were scraped into ice-cold lysis buffer [50 mM Hepes (pH 8.0), 150 mM NaCl, 1% w/v SDS, 1% v/v Triton X-100, 1% v/v IGEPAL CA-630, 1% v/v TWEEN-20, 1% w/v sodium deoxycholate, Pierce Universal Nucleases (20 U/μl; Thermo Fisher Scientific), and 1× cOmplete EDTA-free protease inhibitor cocktail (Roche)]. For the replicate performed using six-well plates, the lysis buffer also contained deubiquitination inhibitors PR-619 (10 μM) and 2-chloroacetamide (40 mM), and the mock-infected sample treated with MG132 was excluded because of contamination of the culture. Lysates were snap-frozen in liquid nitrogen and stored at −70°C.

Protein concentration was estimated by BCA assay (Pierce), and 10 μg of protein was reduced with 10 mM tris(2-carboxyethyl)phosphine and 20 mM 2-chloroacetamide at 80°C for 10 min followed by 15 min for alkylation at RT. Protein in 40 μl was prepared by SP3 clean-up using a Kingfisher Apex (Thermo Fisher Scientific) with a 96–deep-well magnetic head. Four volumes (160 μl) of methanol containing 100 μg of SP3 beads [a 50:50 mixture of E3 and E7 Sera-Mag SpeedBead carboxylate–modified magnetic particles (Cytiva) washed three times in LC-MS grade water (Honeywell)] was added to each sample to induce aggregation. These beads were “slow” mixed for 5 min, collected, washed three times without release in 1 ml of 80% ethanol (Thermo Fisher Scientific), and digested to release peptides into 150 μl of 20 mM ammonium bicarbonate using 400 ng of trypsin/Lys-C (Promega) for 2 hours at 47°C. At all stages beads were gathered for 3 × 10s. The resulting peptide solution was acidified with 10 μl of 1% v/v trifluoracetic acid and cleared at 500*g* for 5 min.

The predigestion protein equivalent of 500 ng of peptides were injected by a Vanquish Neo HPLC onto a Pepmap Neo C18 trapping cartridge (300 μm by 5 mm) and separated over a 0.8 μl/min 23-min 3 to 41% “B” gradient (A: 0.1% formic acid, B: 80% v/v acetonitrile, 0.1% formic acid), with a PepMap RSLC C18 150 μm by 15 cm, 2 μm, 100-Å EasySpray column heated to 50°C. Peptides were characterized by Orbitrap Astral mass spectrometer by electrospray ionization at 1.9 kV over a 380- to 980-*m/z* scan range, RF lens 40%, ion transfer tube temperature of 290°C, 5 × 10^6^ AGC target, 5-ms max ion injection time, at 240-k Orbitrap resolution with FAIMS Pro set to a CV of −48 V. Data independent acquisition with the Astral analyzer used 2-Th optimized windows, 25% HCD collision energy, 5 × 10^4^ AGC target, and 3-ms max ion injection time, over a 150- to 2000-*m/z* scan range.

Raw data were processed by DIA-NN version 2.6.0 (*131*) using a spectral library built from a curated HSV-1 strain KOS proteome and the human proteome (UP000005640, accessed on 28.03.2025) with one missed trypsin/p cleavage, one variable modification of oxidized methionine, N-terminal M excision, N-terminal acetylation, fixed carbamidomethyl C, peptide length 7 to 30, precursor charge 1 to 4, precursor *m/z* 380 to 980, fragment *m/z* 150 to 2000. MS1 and MS2 accuracies were set to 4 and 10, respectively, with 1% protein FDR, match between runs, QuantUMS (precision) and all other default settings used. DIA-NN data were preprocessed in R by removing common contaminant proteins and applying a log_2_ transformation, with removed viral proteins retained separately for analysis (Software S5). Data were then analyzed using Perseus (*124*), and proteins not identified in all conditions across both replicates were removed.

### Calcium ion flux imaging

Day 3 i3Neurons (2.5 × 10^4^) were seeded per well in a PLO-coated 96-well plate, with three technical triplicate wells for each infection condition and time point. After 48 hours, 100 μl of medium was removed and 100 μl of Neuroburst Orange Lentivirus (Sartorius), diluted 1:100 in CN medium, was added. At 24 hpi, the inoculum was replaced with fresh CN medium and, subsequently, half-medium changes were performed thrice weekly. At day 20, the plate was transferred to an Incucyte SX5 for daily imaging with movie mode (2 min per well, 3 images/s, 4× lens). Movie analysis was performed using the Neuronal Activity Analysis software with the following parameters: object size, 100 μm; min cell width, 4 μm; sensitivity, −2; edge split sensitivity, −15; min burst intensity, 0.05. At day 40, Neuroburst-transduced i3Neurons were synchronously infected (MOI 5) with WT, ΔpUL56, or pUL56 AAXA123 HSV-1, or mock-infected. After 1 hour, cells were washed thrice with PBS before being overlaid with conditioned media. To prevent bleaching of the calcium biosensor, wells were scanned only once during the 48-hour infection time course, either at 12, 24, or 48 hpi. 1 μM TTX was added at 50 hpi to wells initially scanned at 12 hpi, which were rescanned an hour later. Wells at 24 hpi were fixed and stained for ICP4 to measure percentage infection as described in (*27*).

### MEA analysis

Wells of a 24-well MEA cytoview plate (Axion Biosystems) were coated with PLO before being seeded with 4 × 10^5^ day 3 i3Neurons. Neurons were matured with half-media changes thrice weekly. From day 14, electrical activity was recorded once every weekday on a Maestro Edge system (Axion Biosystems) with the control software Navigator (Axion Biosystems). The threshold for spike detection was six times the SD of the background signal. Before recordings, plates were loaded into the instrument and equilibrated for 15 min at 37°C, 5% CO_2_. Data were processed with Navigator and activity metrics calculated with Neural Metrics tool (Axion Biosystems). Statistical analysis was performed using Prism 7 (GraphPad), comparing conditions via one-way analysis of variance (ANOVA) and Sidak’s multiple comparisons test.

## Supplementary Materials

### The PDF file includes:

Figs. S1 to S11 Tables S1 to S3 Legends for movies S1 to S3 Legends for data S1 to S5 Software S1 to S5 References
