# Supplementary material for: Herpes simplex virus pUL56 abolishes neuronal activity by removing voltage-gated ion channels from the plasma membrane
Source: Sci Adv. 2026 Oct 7;12(41):eaei1796. doi: 10.1126/sciadv.aei1796 (PMC13644772; doi:10.1126/sciadv.aei1796)
Supplement: Supplementary file 1 [file sciadv.aei1796_sm.pdf]

Science Advances
AAAS

Supplementary Materials for

Herpes simplex virus pUL56 abolishes neuronal activity by removing voltage-gated ion channels from the plasma membrane

Daniel A. Nash *et al.*

Corresponding author: Janet E. Deane, jed55@cam.ac.uk; Stephen C. Graham, scg34@cam.ac.uk

Sci. Adv. **12**, eaei1796 (2026)
DOI: 10.1126/sciadv.aei1796

The PDF file includes:

Figs. S1 to S11
Tables S1 to S3
Legends for movies S1 to S3
Legends for data S1 to S5
Software S1 to S5
References

Other Supplementary Material for this manuscript includes the following:

Movies S1 to S3
Data S1 to S5

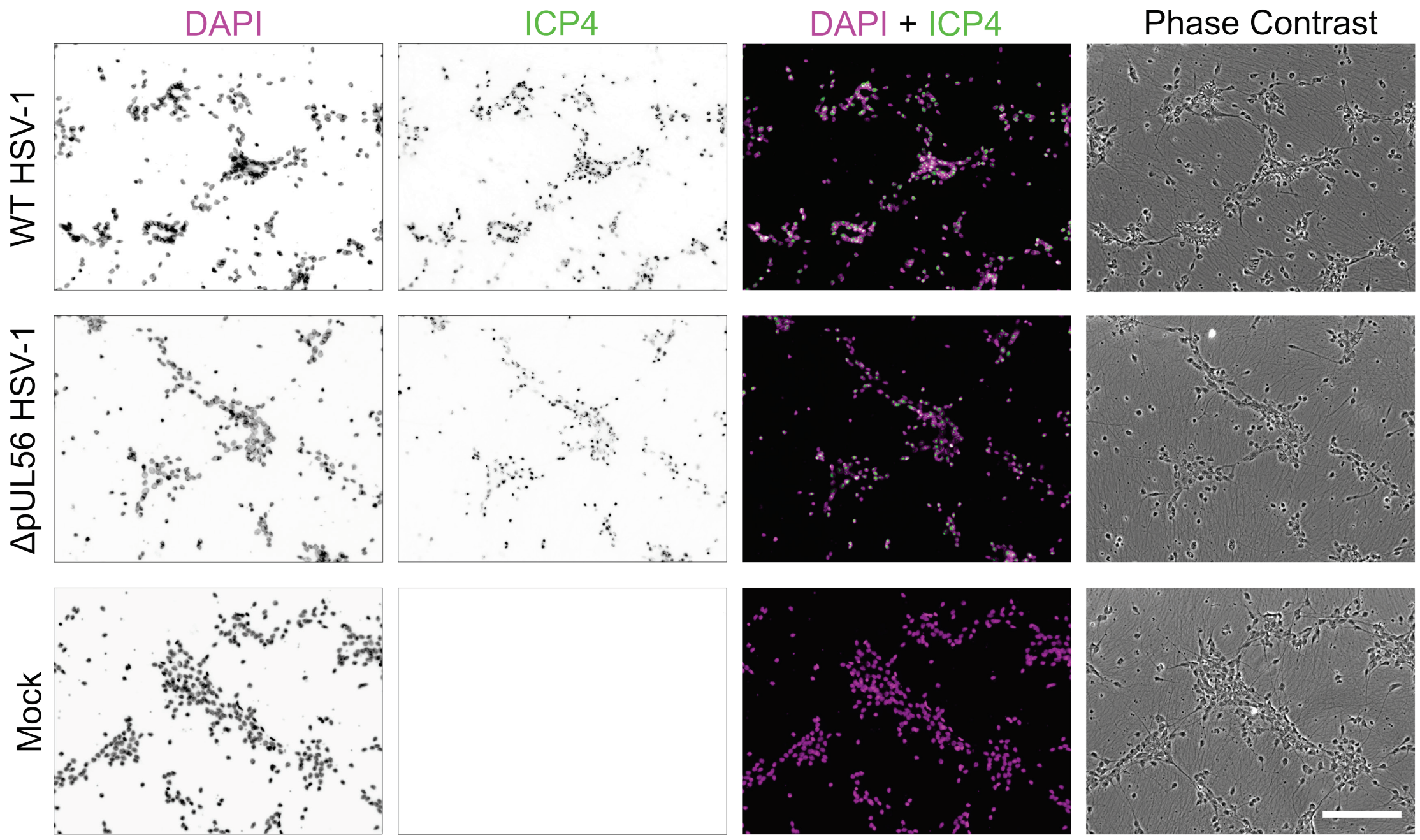

Fig. S1. i3Neurons synchronously infected with WT and ΔpUL56 HSV-1.

i3Neurons were infected at MOI 5 with WT and ΔpUL56 HSV-1 concurrently during sample preparation for temporal proteomics. Cells were fixed 18 hpi, immunostained for ICP4 (green) and stained with DAPI (magenta) to visualize DNA. Co-localization of ICP4 and DAPI signal confirms near 100% infection. Scale bar = 150 µm. Images are representative of three biological experiments.

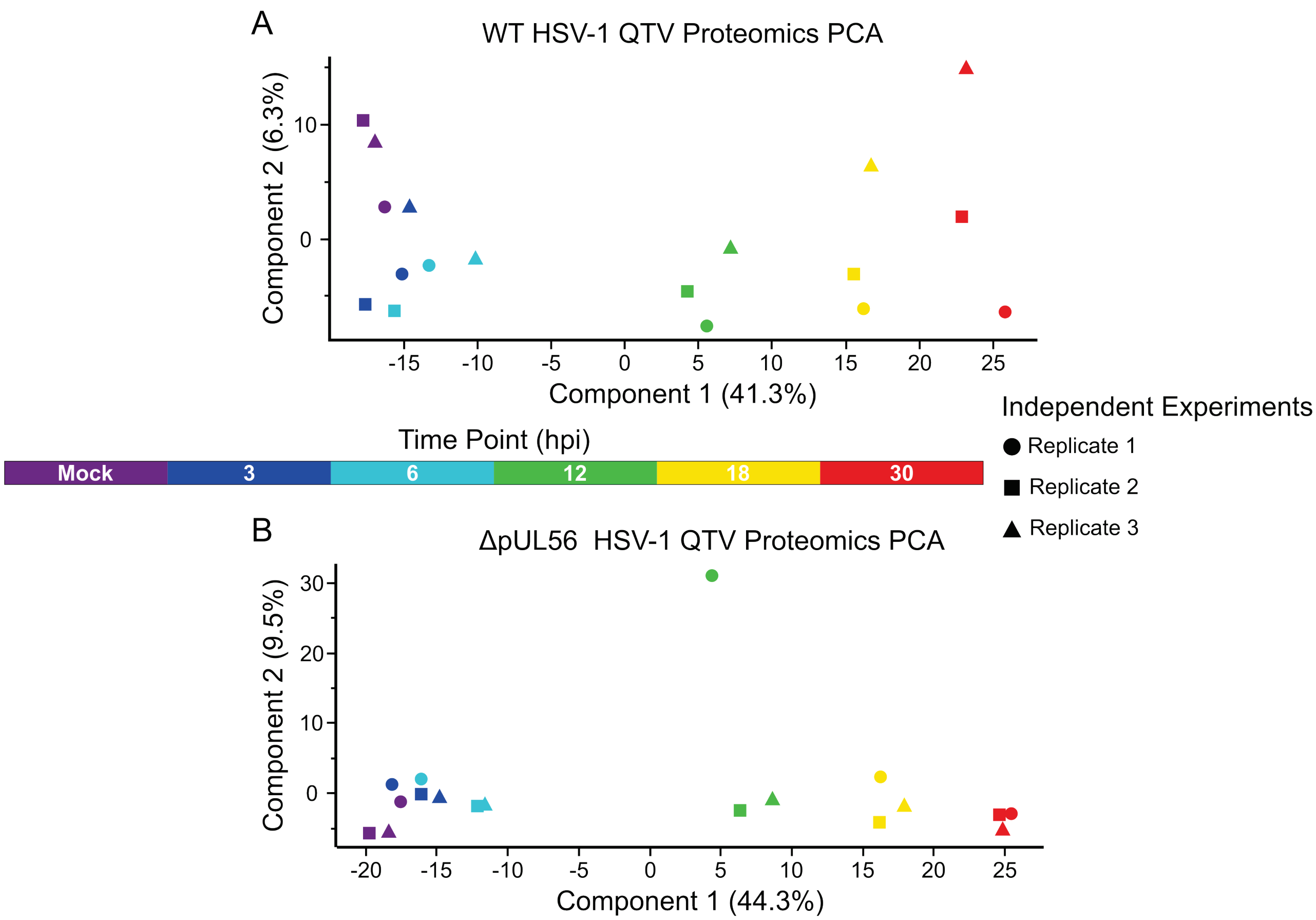

Fig. S2. Principal component analysis (PCA) of temporal proteomics.

PCA for (***A***) WT and (***B***) ΔpUL56 HSV-1 infection experiments are show, each with their corresponding mock infection. Data points are colored by time post infection: 3 h (blue), 6 h (cyan), 12 h (green), 18 h (yellow), 30 h (red), and mock infection (purple). Data points cluster by treatment, not biological replicate (squares, circles and triangles). Separation along the horizontal axis for both experiments represents the largest source of variation, corresponding to time post-infection.

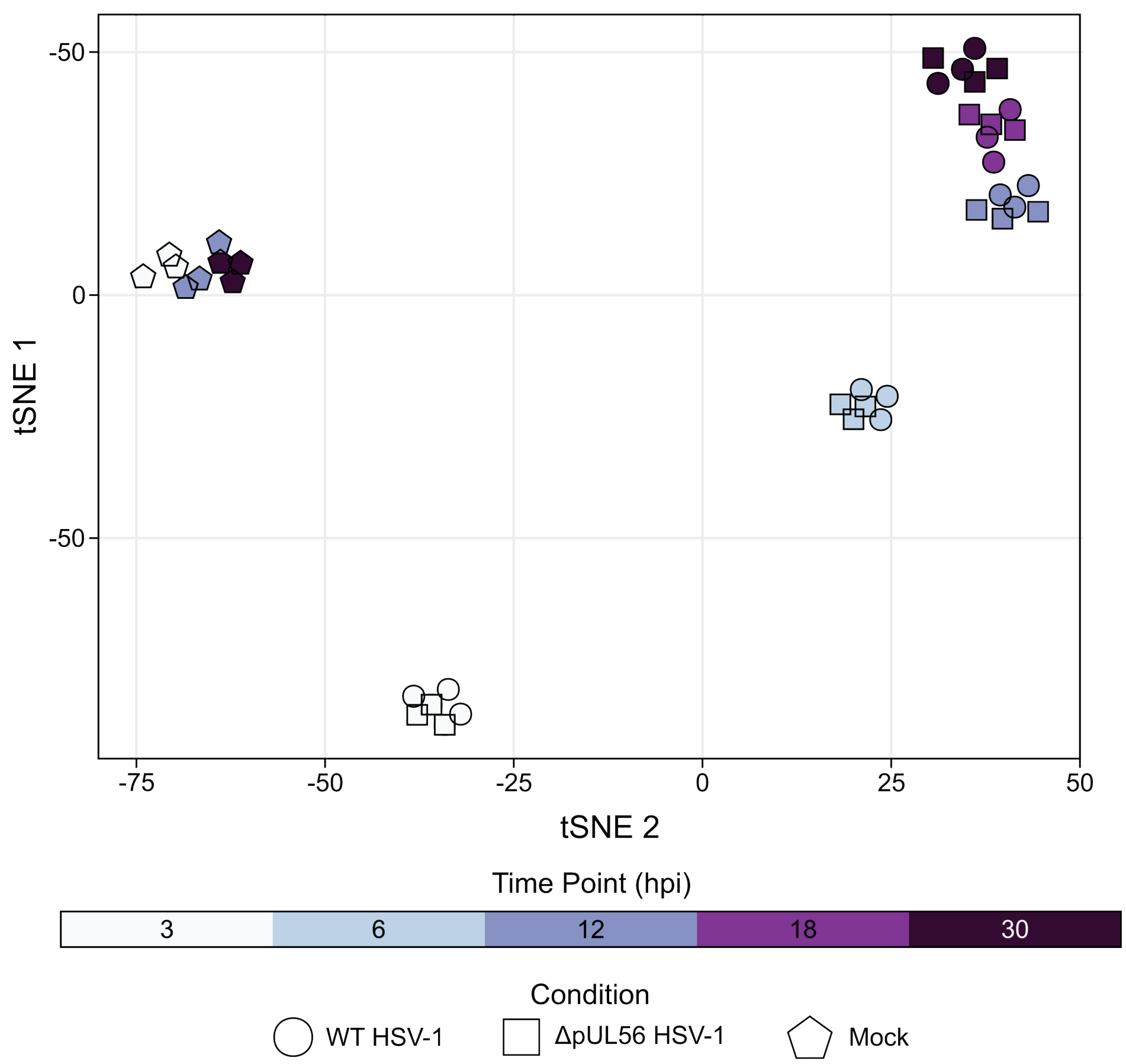

Fig. S3. T-distributed Stochastic Neighbor Embedding (tSNE) analysis of temporal transcriptomics of WT and ΔpUL56 HSV-1 infected i3Neurons.

tSNE analysis was performed on variance-stabilized gene counts, colored by time post (mock)-infection: 3 h (light blue), 6 h (blue), 12 h (slate blue), 18 h (light purple), 30 h (deep purple). Data clusters by treatment and not by biological replicate. At each timepoint WT and ΔpUL56 infection experiments clustering together, consistent with both viruses causing similar changes to the transcriptome of infected i3Neurons.

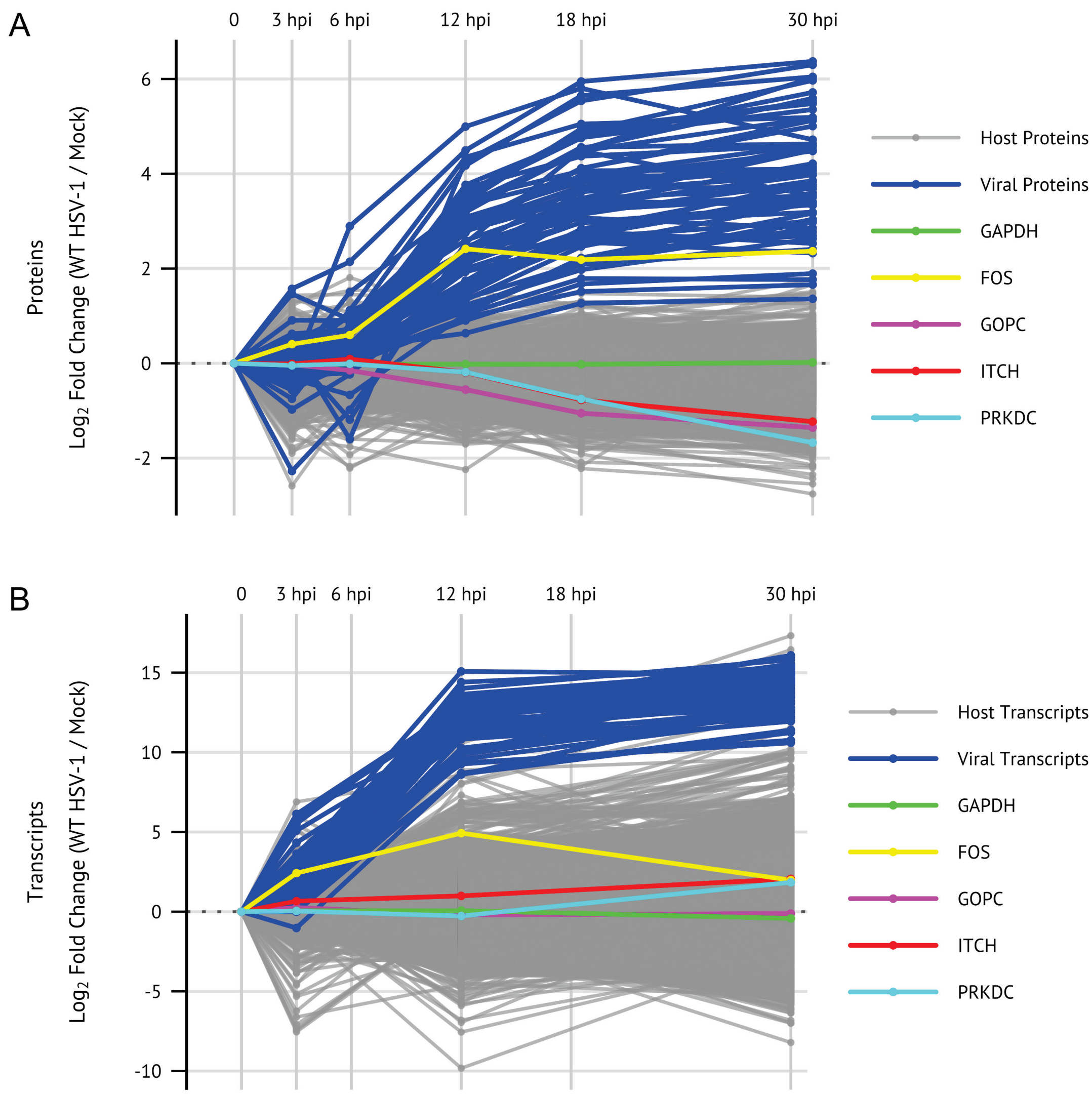

Fig. S4. Temporal changes in host and virus protein and RNA transcript abundance following infection of i3Neurons with WT HSV-1.

The mean $\log_2$ fold change in abundance of each (***A***) protein and (***B***) transcript is plotted as a function of time post-infection, with mock-infected samples displayed as time 0. Cellular gene products are plotted in grey and viral gene products in blue. (***A***) Log_2 fold change is calculated relative to the mock-infected sample (taken at 12 hpi). Targets of virus-mediated degradation are highlighted (GOPC, magenta; PRKDC, cyan; ITCH, red), as is cFOS (yellow) that increases in abundance during infection. The abundance of GAPDH (green) does not change during infection. (***B***) Log_2 fold change is calculated relative to the mock-infected samples taken at the given time points; the 6 hpi and 18 hpi are excluded because mock samples were not taken at those time points. Selected genes are colored as in (*A*). The global decline in mRNA abundance caused by host shutoff is not captured in these plots owing to DESeq2 normalization that assumes constant total mRNA levels, as discussed in (*135*).

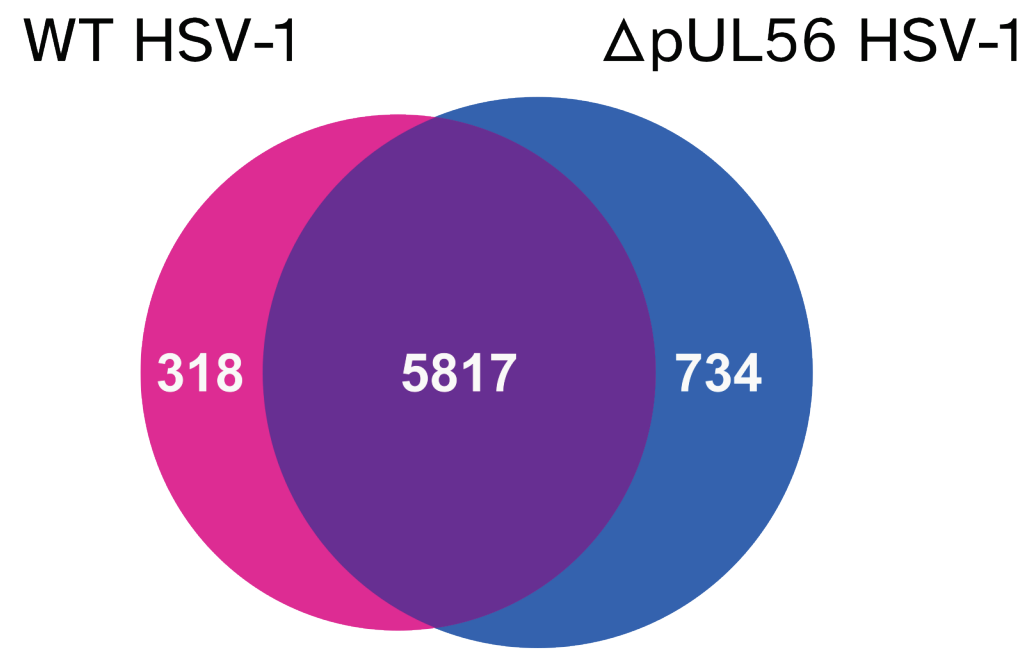

Fig. S5. Unique protein groups identified in temporal proteomics of i3Neurons infected with WT or ΔpUL56 HSV-1.

Protein groups can represent more than one protein or isoform if all the identified constituent peptides cannot be unambiguously assigned to a single protein or isoform (*122*). 5817 protein groups were identified in both experiments, whilst 318 and 734 protein groups were identified only in the WT -1 and ΔpUL56 HSV-1 proteomics analyses, respectively.

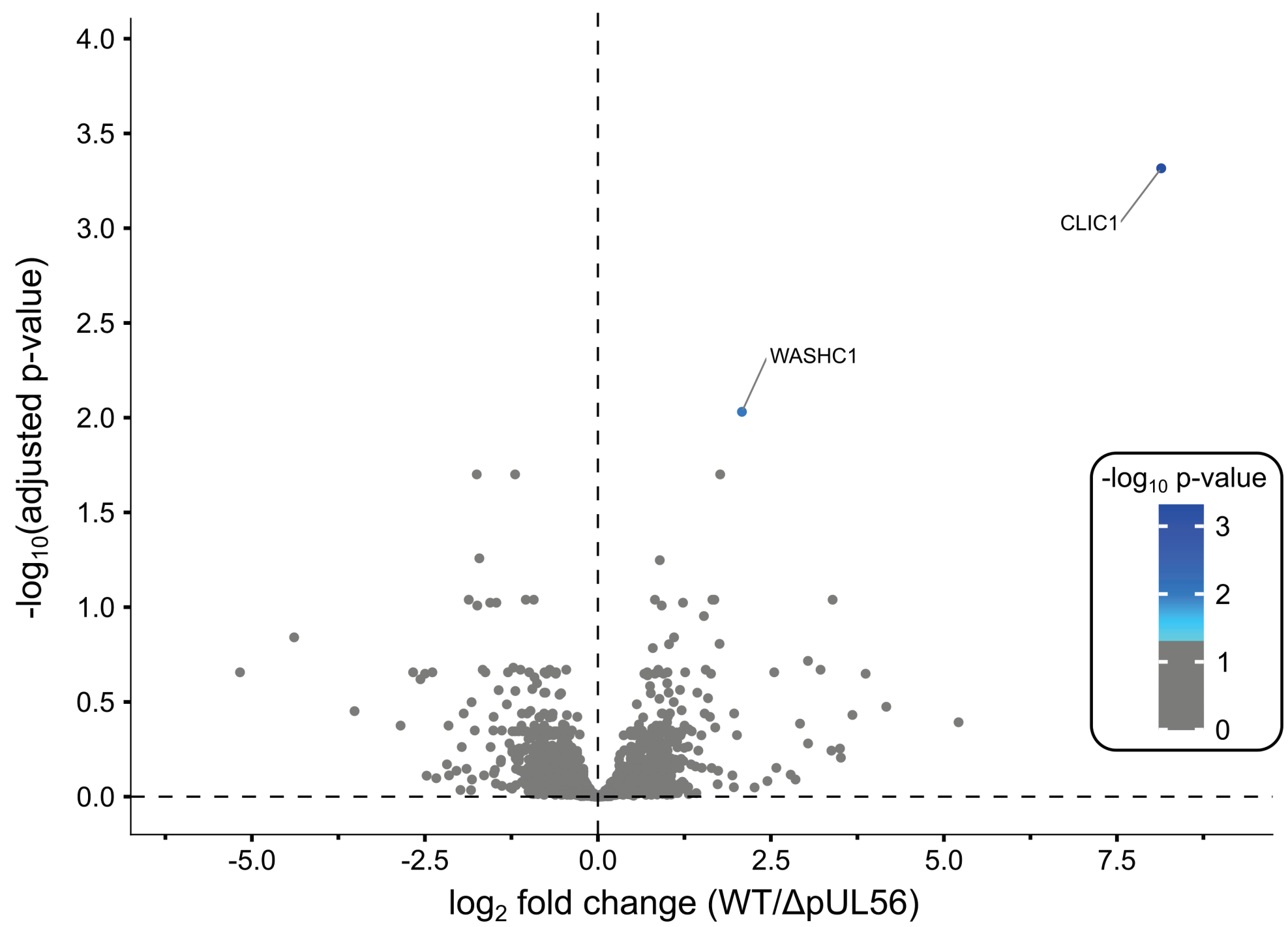

Fig. S6. Changes in i3Neuron transcript abundance in WT versus ΔpUL56 HSV-1 infected i3Neurons at 30 hpi.

Horizontal axis shows average $\log_2$ fold change and vertical axis shows FDR-adjusted significance for three independent experiments. Significantly altered transcripts ($\log_2$ fold change ≥ 2 and $p \leq 0.05$) labelled and colored by significance (blue).

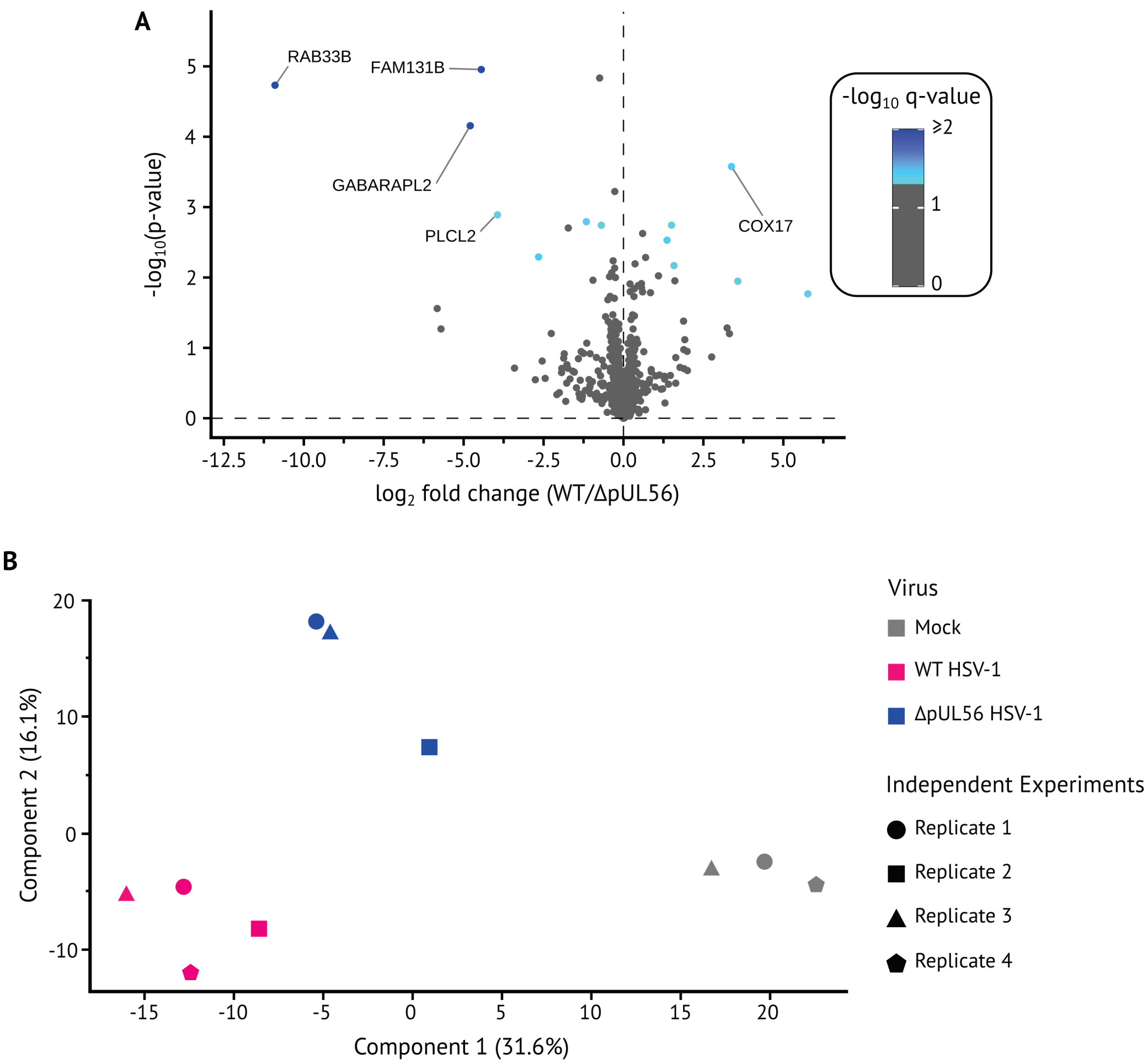

Fig. S7. i3Neuron HSV-1 infection PM proteomic analysis.

(***A***) Change in abundance of proteins in WT versus ΔpUL56 HSV-1 infection (24 hpi) that were identified after PM protein biotinylation and streptavidin capture, where the proteins lack GO cellular compartment annotations "plasma membrane", "cell surface", or "extracellular region". Horizontal axis shows average fold change and vertical axis shows significance (two-sided t-test) for three independent experiments. Proteins are colored by FDR-corrected significance. 716 proteins were identified after filtering (identification in three replicates of at least one infection condition) and the majority of these are presumed to be proteins that bind non-specifically to the streptavidin resin, although they may also represent cytoplasmic proteins that co-purify with biotinylated proteins. Selected proteins with significantly altered abundance are labelled. (***B***) PCA was performed on protein abundances that had been log_2-transformed, filtered to retain only GO cellular compartment annotations "plasma membrane", "cell surface", or "extracellular region", then processed to impute missing values. Samples cluster by treatment (mock-infection, grey; WT HSV-1 infection, magenta; ΔpUL56 HSV-1 infection, blue) rather than by biological replicate (circles, squares, triangles and pentagons).

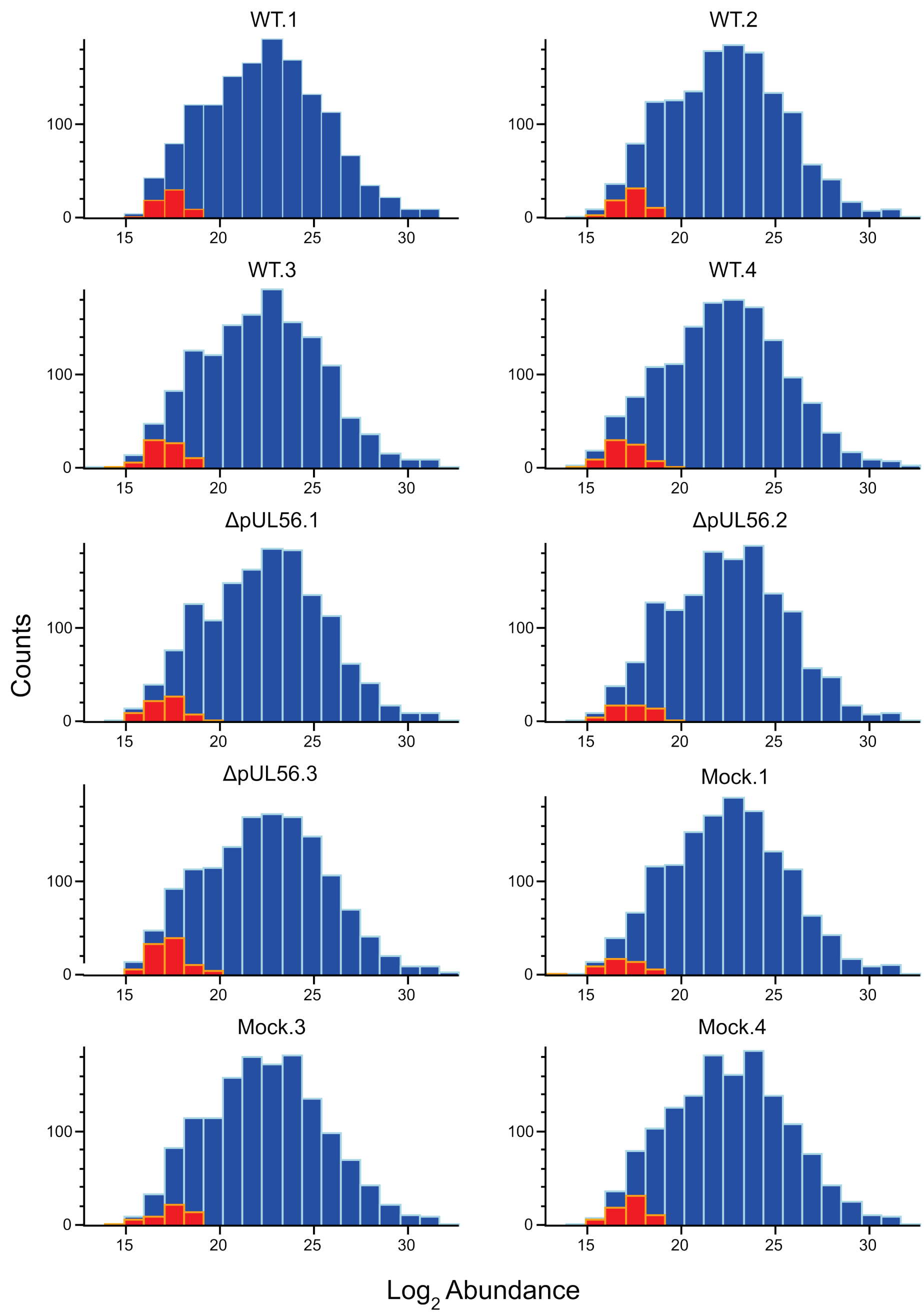

Fig. S8. PM-enriched protein abundances per sample approximately follow a Gaussian distribution after imputation.

Frequency histogram of observed (blue) and imputed (red) MaxLFQ abundances, log_2 transformed, plotted per individual mass spectrometry experiment as shown.

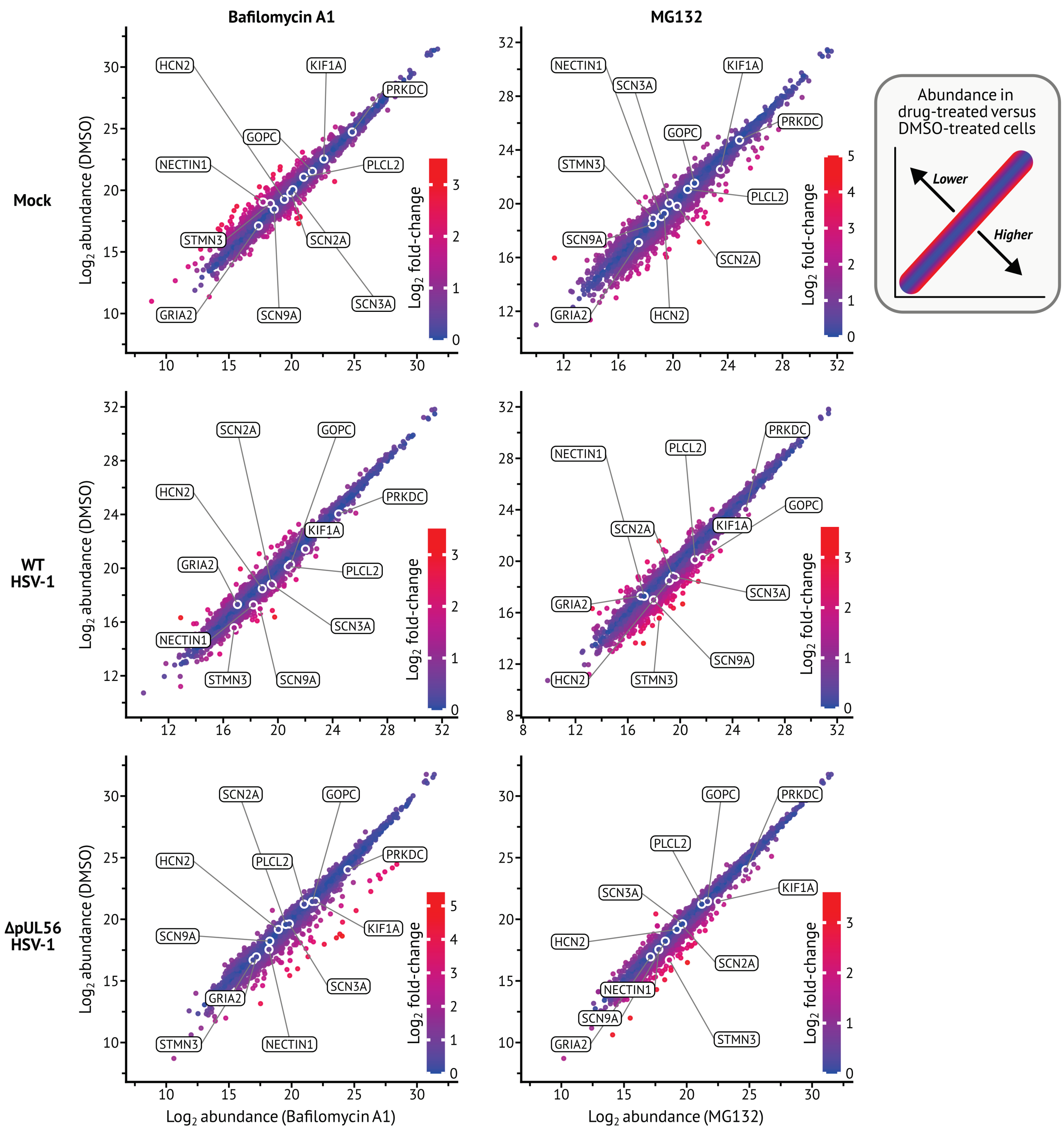

Fig. S9. Protein abundance in proteolysis inhibitor versus DMSO treated cells infected with HSV-1.

Average $\log_2$ QuantUMS abundance in DMSO (control) versus bafilomycin A1 (left) or MG132 (right) treated cells that were mock-infected (top) or infected with WT (middle) or ΔpUL56 (bottom) HSV-1. Data shown are from two independent experiments. Proteins are colored by

average log_2 fold change. Proteins below the diagonal represent those with higher abundance (rescued) following drug treatment, while those above the diagonal are less abundant in drug treated cells.

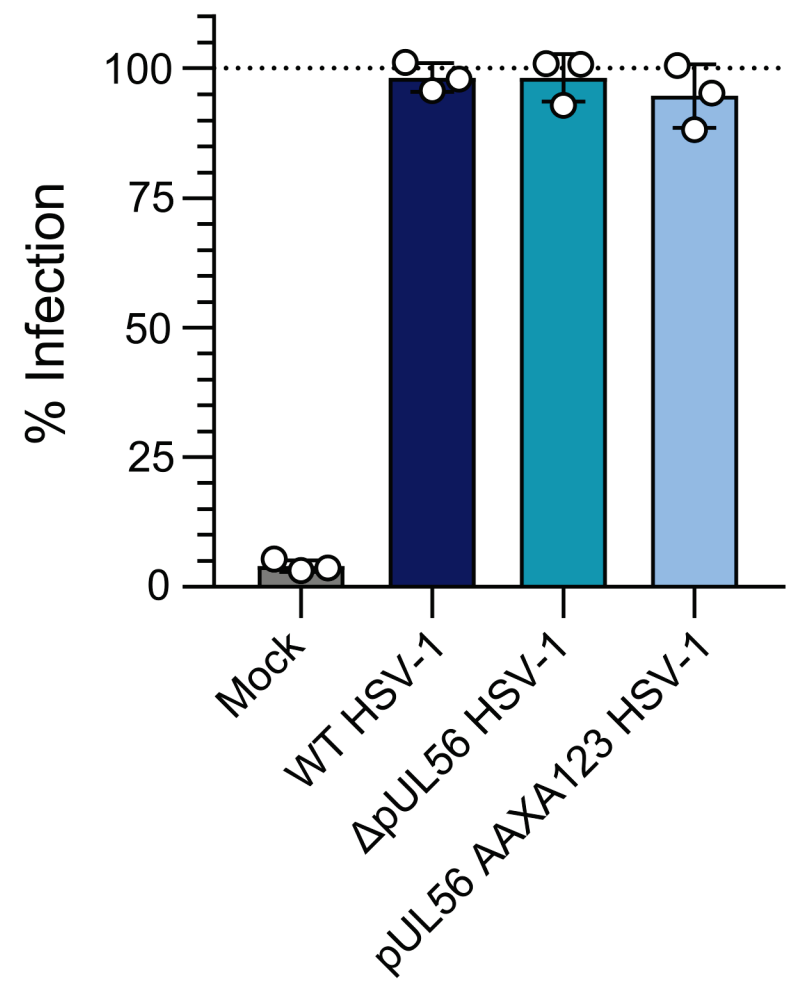

Fig. S10. i3Neurons were infected with HSV-1 at near-100% efficiency for calcium-flux kinetics assays.

i3Neurons that had been used for calcium-flux kinetic imaging at 24 hpi were then fixed, immunostained for ICP4 and stained with propidium iodide to visualize HSV-1 infection and DNA, respectively. Co-localization was quantitated as proportion of propidium iodide positive nuclei with ICP4 staining and is shown as mean ± SD from technical triplicate measurements for one of the calcium-flux kinetics assay biological replicates (24 hpi). For all viruses, approximately 100% of i3Neurons are infected.

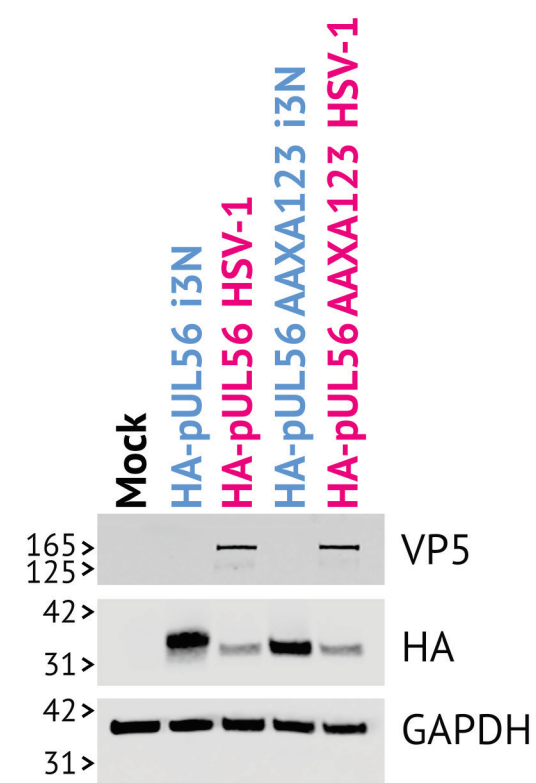

Fig. S11. Relative expression HA-pUL56 in infected versus stably transfected i3Neurons. Immunoblot comparing HA-pUL56 expression in day 15 i3Neurons that had been mock infected (black label) or infected (MOI 5) with HA-pUL56 or HA-pUL56 AAXA123 HSV-1 (pink labels) for 24 h, versus day 15 i3Neurons stably transfected with HA-pUL56 or HA-pUL56 AAXA123 (blue labels). VP5 is shown as an HSV-1 infection control and GAPDH as a loading control. Data is representative of one biological experiment.

Table S1. Oligonucleotide primers used in this study.

Primer	Sequence (5′ to 3′)	Notes
GOPC KD1 fwd	CACCGACAGAACCGCAGGA GTAACG	Targets GOPC Exon 1 (nt 86–105, antisense), with added BpiI site
GOPC KD1 rev	AAACCGTTACTCCTGCGGTT CTGTC	Targets GOPC Exon 1 (nt 86–105, antisense), with added BpiI site
GOPC KD2 fwd	CACCGCTGGCGGGGTATCC ATGTTC	Targets GOPC Exon 1 (nt 328–347, sense), with added BpiI site
GOPC KD2 rev	AAACGAACATGGATACCCCG CCAGC	Targets GOPC Exon 1 (nt 328–347, sense), with added BpiI site
iC10_PB_Internal_FWD	CTGGCGAAAGGGGGATGTG	NEBuilder HiFi assembly of HA-pUL56 piggyBac expression vector
iC10_PB_Internal_REV	CACATCCCCCTTTCGCCAG	NEBuilder HiFi assembly of HA-pUL56 piggyBac expression vector
Frag1_HA-UL56_FWD	CTCGAGCCACCATGTACCCA TACGATGTTCCAGATTACGCT GCTTCGGAGGCGGCG	NEBuilder HiFi assembly of HA-pUL56 piggyBac expression vector
Frag1_HA-UL56_REV	CGTATGGGTACATGGTGGCT CGAGCGGCCGCCGTCGACA TTCTTTGCCAAAATG	NEBuilder HiFi assembly of HA-pUL56 piggyBac expression vector
UL56_Frag2_FWD	CTGTGGCGGTGAGAATT CCTTGGACGCGTCTGCAG	NEBuilder HiFi assembly of HA-pUL56 piggyBac expression vector
UL56_Frag2_REV	CAAGGAATTCTCACCGCC ACAGGAATACCAG	NEBuilder HiFi assembly of HA-pUL56 piggyBac expression vector
Xho1_HA-UL56_FWD	GCGGCCGCTCGAGATGTAC CCATACGATGTTCCAGATTA CGCTGCTTCGGAGGCGGCG	Subcloning pUL56 AAXA123 into piggyBac expression vector
UL56_Frag2_REV	CTGCAGACGCGTCCAAGGA ATTCTCACCGCCACAGGAAT ACCAG	Subcloning pUL56 AAXA123 into piggyBac expression vector
COL630	ACCTAGCGCGCGGGAGTCG TGGCTTTGGGGCGCATCCAT GTACCCATACGACGTCCCCG ACTACGCTTCGGAGGCGGAG GATGACGACGATAAGTAGGG	Forward primer for insertion of a HA tag at N terminus of pUL56. Generates an NdeI site.
COL631	CGCTCCATAGACCCGCGTC GGGTTGCGCCGCCTCCGAA GCGTAGTCGGGGACGTCGT ATGGGTACATGGATGCGCCC CAACCAATTAACCAATTCTGA TTAG	Reverse primer for insertion of a HA tag at N terminus of pUL56. Generates an NdeI site.

Table S2. TMT sample labelling
Independent mock infections used in the WT and ΔpUL56 HSV-1 proteomics experiments.

Experiment	Infection	Time post-infection (h)	Biological replicate	18-plex TMTpro label
**WT HSV-1**	WT	3	1	126C
	WT	6	1	127N
	WT	12	1	127C
	WT	18	1	128N
	WT	30	1	128C
	WT	3	2	129N
	WT	6	2	129C
	WT	12	2	130N
	WT	18	2	130C
	WT	30	2	131N
	Mock	12	1	131C
	Mock	12	2	132N
	WT	3	3	132C
	WT	6	3	133N
	WT	12	3	133C
	WT	18	3	134N
	WT	30	3	134C
	Mock	12	3	135N
**ΔpUL56 HSV-1**	ΔpUL56	3	1	126C
	ΔpUL56	6	1	127N
	ΔpUL56	12	1	127C
	ΔpUL56	18	1	128N
	ΔpUL56	30	1	128C
	ΔpUL56	3	2	129N
	ΔpUL56	6	2	129C
	ΔpUL56	12	2	130N
	ΔpUL56	18	2	130C
	ΔpUL56	30	2	131N
	Mock	12	1	131C
	Mock	12	2	132N
	ΔpUL56	3	3	132C
	ΔpUL56	6	3	133N
	ΔpUL56	12	3	133C
	ΔpUL56	18	3	134N
	ΔpUL56	30	3	134C
	Mock	12	3	135N

Table S3. TMT-Pro 18-plex reporter ion isotopic impurity values.
NA, not applicable.

Mass Tag	Reporter Ion Mass	-2		-1		M+	+1		+2	
		-2× 13C	-13C -15N	-13C	-15N		+15N	+13C	+15N +13C	+2× 13C
**TMTpro-126**	126.127726	NA	NA	NA	NA	100%	0.0034	9.31% (127C)	0.02%	0.32%
**TMTpro-127N**	127.124761	NA	NA	NA	0.78% (126)	100%	NA	9.41% (128N)	NA	0.33%
**TMTpro-127C**	127.131081	NA	NA	0.93% (126)	NA	100%	0.0035	8.63% (128C)	0.01%	0.27%
**TMTpro-128N**	128.128116	NA	0.00%	0.95% (127N)	0.0079	100%	NA	8.38% (129N)	NA	0.26%
**TMTpro-128C**	128.134436	0.00%	NA	1.47% (127C)	NA	100%	0.0034	6.91% (129C)	0	0.15%
**TMTpro-129N**	129.131471	0.00%	0.00%	1.46% (128N)	0.0128	100%	NA	6.86% (130N)	NA	0.15%
**TMTpro-129C**	129.13779	0.51%	NA	2.74% (128C)	NA	100%	0.0036	6.15% (130C)	0	0.11%
**TMTpro-130N**	130.134825	0.49%	0.02%	2.76% (129N)	0.0062	100%	NA	5.98% (131N)	NA	0.11%
**TMTpro-130C**	130.141145	0.04%	NA	3.10% (129C)	NA	100%	0.0042	4.82% (131C)	0.02%	0.06%
**TMTpro-131N**	131.13818	0.04%	0.04%	3.09% (130N)	0.0136	100%	NA	4.75% (132N)	NA	0.06%
**TMTpro-131C**	131.1445	0.08%	NA	3.81% (130C)	NA	100%	0.004	3.29% (132C)	0.03%	0.03%
**TMTpro-132N**	132.141535	0.04%	0.00%	2.84% (131N)	0.0079	100%	NA	3.51% (133N)	NA	0.02%
**TMTpro-132C**	132.147855	0.11%	NA	4.55% (131C)	NA	100%	0.0043	1.86% (133C)	0	0.00%
**TMTpro-133N**	133.14489	0.36%	0.01%	3.64% (132N)	0.0082	100%	NA	1.94% (134N)	NA	0.00%
**TMTpro-133C**	133.15121	0.22%	NA	4.96% (132C)	NA	100%	0.0034	1.03% (134C)	0	NA
**TMTpro-134N**	134.148245	0.40%	0.04%	4.92% (133N)	0.001	100%	NA	1.05% (135N)	NA	NA
**TMTpro-134C**	134.154565	0.16%	NA	5.81% (133C)	NA	100%	0.39%	NA (135C)	NA	NA
**TMTpro-135N**	135.151600	0.21%	0.04%	5.90% (134N)	0.74%	100%	NA	NA (136N)	NA	NA

Movie S1.

Calcium-flux kinetics imaging of i3Neurons mock-infected or synchronously infected (MOI 5) with wild-type or mutant HSV-1. Movie was recorded at 12 hpi and represents 90 s of imaging. 800 µM scale bar.

Movie S2.

Calcium-flux kinetics imaging of i3Neurons mock-infected or synchronously infected (MOI 5) with wild-type or mutant HSV-1. Movie was recorded at 24 hpi and represents 90 s of imaging. 800 µM scale bar.

Movie S3.

Calcium-flux kinetics imaging of i3Neurons mock-infected or synchronously infected (MOI 5) with wild-type or mutant HSV-1. Movie was recorded at 48 hpi and represents 90 s of imaging. 800 µM scale bar.

Data S1.

Interactive spreadsheet with "plotter" function to explore multi-omics analysis of HSV-1 infected i3Neurons. "QTV" worksheet enables generation of plots showing changes in gene product abundance by typing in the desired gene name, with proteins and transcripts shown on separate graphs as mean ± SD (three biological replicates) connected by solid and dashed lines, respectively. For each gene product, abundance is normalized from zero to the highest observed value. Data for WT and ΔpUL56 infection are pink and blue, respectively. For proteins, the grey values at time 0 represent the mock infection sample harvested at 12 hpi. For transcripts, the three mock infection time points are each shown (grey). "i3Neurone vs Keratinocyte WCP" worksheet shows the $\log_2$ fold change in whole cell proteomics (WCP) of WT HSV-1 vs mock infection for i3Neurons at 30 hpi and keratinocytes at 18 hpi (*17*). "PMP" worksheet shows PM proteomics of i3Neurons at 18 hpi. Top graphs show $\log_2$ transformed MaxLFQ abundance of infected with WT (pink) or ΔpUL56 (blue) HSV-1, or mock-infected (grey). Mean ± SD is also shown for three (mock and ΔpUL56 HSV-1) or four (WT HSV-1) biological replicates. Bottom graphs show average $\log_2$ abundances fold change for WT versus mock, ΔpUL56 versus mock or WT versus ΔpUL56 infection.

Data S2.

Temporal proteomics analysis of HSV-1 infected i3Neurons. Protein groups (UniProt) and gene names are shown. For worksheets "WT HSV-1 versus Mock" and "ΔpUL56 HSV-1 versus Mock", intensity-Based Absolute Quantification (iBAQ) abundance, number of peptides identified, average $\log_2$ fold change (three biological replicates) of infected cell samples harvested at 3, 6, 12, 18 and 30 hpi versus mock infected samples harvested at 12 hpi, and average normalized reporter ion intensities for each sample are shown. For "WT versus ΔpUL56 HSV-1" worksheet, difference in $\log_2$ fold change versus mock is shown for each time point. For all, FDR-corrected significance (q)-values are shown and significant changes are marked.

Data S3.

Temporal transcriptomics analysis of HSV-1 infected i3Neurons. In worksheet "DESqe2 Contrast" the Ensembl gene ID, gene name and Base Mean average normalized count is shown, plus log2 fold change and FDR-adjusted p-value for WT versus mock, ΔpUL56 versus mock and

WT versus ΔpUL56 infection at 3, 12 and 30 hpi. "Transcript Counts" worksheet shows the gene ID and normalized counts per transcripts for each replicate experiment at each time point.

Data S4.

Plasma membrane proteomics analysis of HSV-1 infected i3Neurons at 24 hpi. "PMP" worksheet shows protein (UniProt) and gene name plus average $\log_2$ fold change (three biological replicates) of WT versus mock, ΔpUL56 versus mock, or WT versus ΔpUL56 HSV-1 infection, with FDR-corrected significance (q)-values are shown and significant changes marked. Average MaxLFQ abundances for each sample are also shown.

Data S5.

Whole-cell proteomics analysis at 24 hpi of HSV-1 infected i3Neurons treated with proteolysis inhibitors from 6 hpi. "Cellular Proteins" worksheet shows protein (UniProt), gene name, number of proteotypic sequences, $\log_2$ QuantUMS abundance for each biological replicate of each condition, and average log2 fold change of drug versus DMSO treatment for each condition. "Virus Proteins" worksheet shows gene names, abundances and average $\log_2$ fold changes for virus proteins from infected cell conditions only.

Software S1.

R code for filtering and label normalization of whole cell proteomics data.

Software S2.

R code for DESeq2 analysis of transcriptomics data.

Software S3.

R code for pre-processing of plasma membrane proteomics data.

Software S4.

R code for GO term filtering of plasma membrane proteomics data.

Software S5.

R code for pre-processing of proteolysis inhibitor whole-cell proteomics data.

REFERENCES

1. M. Harfouche, S. AlMukdad, A. Alareeki, A. M. M. Osman, S. Gottlieb, J. Rowley, L. J. Abu-Raddad, K. J. Looker, Estimated global and regional incidence and prevalence of herpes simplex virus infections and genital ulcer disease in 2020: Mathematical modelling analyses. *Sex. Transm. Infect.* **101**, 214–223 (2025).

2. M. P. Nicoll, J. T. Proença, S. Efstathiou, The molecular basis of herpes simplex virus latency. *FEMS Microbiol. Rev.* **36**, 684–705 (2012).

3. R. J. Whitley, Herpes simplex encephalitis: Adolescents and adults. *Antiviral Res.* **71**, 141–148 (2006).

4. M. J. Bradshaw, A. Venkatesan, Herpes simplex virus-1 encephalitis in adults: Pathophysiology, diagnosis, and management. *Neurotherapeutics* **13**, 493–508 (2016).

5. A. Venkatesan, B. D. Michael, J. C. Probasco, R. G. Geocadin, T. Solomon, Acute encephalitis in immunocompetent adults. *Lancet* **393**, 702–716 (2019).

6. L. K. Jørgensen, L. S. Dalgaard, L. J. Østergaard, M. Nørgaard, T. H. Mogensen, Incidence and mortality of herpes simplex encephalitis in Denmark: A nationwide registry-based cohort study. *J. Infect.* **74**, 42–49 (2017).

7. L. S. Duerlund, L. Larsen, M. Storgaard, H. Mens, L. Wiese, M. P. G. Jepsen, B. R. Hansen, H. R. Lüttichau, C. Ø. Andersen, M. Blaabjerg, A. Venkatesan, H. Nielsen, J. Bodilsen, DASGIB study group, Herpes simplex virus type 1 encephalitis: A prospective population-based cohort study. *Clin. Infect. Dis.* **82**, e1384–e1392 (2026).

8. R. J. Whitley, S. J. Soong, R. Dolin, G. J. Galasso, L. T. Ch'ien, C. A. Alford, Adenine arabinoside therapy of biopsy-proved herpes simplex encephalitis. National Institute of Allergy and Infectious Diseases collaborative antiviral study. *N. Engl. J. Med.* **297**, 289–294 (1977).

9. N. D. Rocha, S. K. de Moura, G. A. B. da Silva, R. Mattiello, D. K. Sato, Neurological sequelae after encephalitis associated with herpes simplex virus in children: Systematic review and meta-analysis. *BMC Infect. Dis.* **23**, 55 (2023).

10. J. P. Stahl, A. Mailles, T. De Broucker, Steering Committee and Investigators Group, Herpes simplex encephalitis and management of acyclovir in encephalitis patients in France. *Epidemiol. Infect.* **140**, 372–381 (2012).

11. P. Jain, A. Jain, A. Kumar, S. Prakash, D. N. Khan, K. P. Singh, R. K. Garg, R. Kumar, G. A. Kumar, Epidemiology and etiology of acute encephalitis syndrome in North India. *Jpn. J. Infect. Dis.* **67**, 197–203 (2014).

12. K. Lopatko Lindman, E.-S. Hemmingsson, B. Weidung, J. Brännström, M. Josefsson, J. Olsson, F. Elgh, P. Nordström, H. Lövheim, Herpesvirus infections, antiviral treatment, and the risk of dementia-a registry-based cohort study in Sweden. *Alzheimers Dement.* **7**, e12119 (2021).

13. K. Araya, R. Watson, K. Khanipov, G. Golovko, G. Taglialatela, Increased risk of dementia associated with herpes simplex virus infections: Evidence from a retrospective cohort study using U.S. electronic health records. *J. Alzheimer's Dis.* **104**, 393–402 (2025).

14. Y. Liu, C. Johnston, N. Jarousse, S. P. Fletcher, S. Iqbal, Association between herpes simplex virus type 1 and the risk of Alzheimer’s disease: A retrospective case-control study. *BMJ Open* **15**, e093946 (2025).

15. R. F. Itzhaki, Corroboration of a major role for herpes simplex virus type 1 in Alzheimer’s disease. *Front. Aging Neurosci.* **10**, 324 (2018).

16. D. M. Cairns, N. Rouleau, R. N. Parker, K. G. Walsh, L. Gehrke, D. L. Kaplan, A 3D human brain-like tissue model of herpes-induced Alzheimer’s disease. *Sci. Adv.* **6**, eaay8828 (2020).

17. T. K. Soh, C. T. R. Davies, J. Muenzner, L. M. Hunter, H. G. Barrow, V. Connor, C. R. Bouton, C. Smith, E. Emmott, R. Antrobus, S. C. Graham, M. P. Weekes, C. M. Crump, Temporal proteomic analysis of herpes simplex virus 1 infection reveals cell-surface remodeling via pUL56-mediated GOPC degradation. *Cell Rep.* **33**, 108235 (2020).

18. T. Hennig, L. Djakovic, L. Dölken, A. W. Whisnant, A review of the multipronged attack of herpes simplex virus 1 on the host transcriptional machinery. *Viruses* **13**, 1836 (2021).

19. K. M. Scherer, J. D. Manton, T. K. Soh, L. Mascheroni, V. Connor, C. M. Crump, C. F. Kaminski, A fluorescent reporter system enables spatiotemporal analysis of host cell modification during herpes simplex virus-1 replication. *J. Biol. Chem.* **296**, 100236 (2021).

20. P. N. Canova, A. J. Charron, D. A. Leib, Models of herpes simplex virus latency. *Viruses* **16**, 747 (2024).

21. H. Fu, D. Pan, Mechanisms of HSV gene regulation during latency and reactivation. *Virology* **602**, 110324 (2025).

22. M. T. Hussain, B. A. Stanfield, D. I. Bernstein, Small animal models to study herpes simplex virus infections. *Viruses* **16**, 1037 (2024).

23. A. Rybak-Wolf, E. Wyler, T. M. Pentimalli, I. Legnini, A. Oliveras Martinez, P. Glažar, A. Loewa, S. J. Kim, B. B. Kaufer, A. Woehler, M. Landthaler, N. Rajewsky, Modelling viral encephalitis caused by herpes simplex virus 1 infection in cerebral organoids. *Nat. Microbiol.* **8**, 1252–1266 (2023).

24. C. Wang, M. E. Ward, R. Chen, K. Liu, T. E. Tracy, X. Chen, M. Xie, P. D. Sohn, C. Ludwig, A. Meyer-Franke, C. M. Karch, S. Ding, L. Gan, Scalable production of iPSC-derived human neurons to identify Tau-lowering compounds by high-content screening. *Stem Cell Reports* **9**, 1221–1233 (2017).

25. M. S. Fernandopulle, R. Prestil, C. Grunseich, C. Wang, L. Gan, M. E. Ward, Transcription factor-mediated differentiation of human iPSCs into neurons. *Curr. Protoc. Cell Biol.* **79**, e51 (2018).

26. A. H. M. Ng, P. Khoshakhlagh, J. E. Rojo Arias, G. Pasquini, K. Wang, A. Swiersy, S. L. Shipman, E. Appleton, K. Kiaee, R. E. Kohman, A. Vernet, M. Dysart, K. Leeper, W. Saylor, J. Y. Huang, A. Graveline, J. Taipale, D. E. Hill, M. Vidal, J. M. Melero-Martin, V. Busskamp, G. M. Church, A comprehensive library of human transcription factors for cell fate engineering. *Nat. Biotechnol.* **39**, 510–519 (2021).

27. D. A. Nash, A. S. Nicholson, H. G. Barrow, V. Connor, C. M. Crump, J. E. Deane, S. C. Graham, Optimization of lytic herpes simplex virus infection in human induced pluripotent stem cell-derived cortical neurones. *J. Gen. Virol.* **107**, 002237 (2026).

28. H. S. Oh, S.-F. Chou, P. Raja, J. Shim, B. Das, J. M. Pesola, N. Romero, J. S. Lee, A. Ng, E. D. Buttermore, G. Church, C. J. Woolf, D. M. Coen, D. M. Knipe, Validation of human sensory neurons derived from inducible pluripotent stem cells as a model for latent infection and reactivation by herpes simplex virus 1. *MBio* **16**, e0187125 (2025).

29. Y. Deng, Y. Lin, S. Chen, Y. Xiang, H. Chen, S. Qi, H. S. Oh, B. Das, G. Komazin-Meredith, J. M. Pesola, D. M. Knipe, D. M. Coen, D. Pan, Neuronal miR-9 promotes HSV-1 epigenetic silencing and latency by repressing Oct-1 and Onecut family genes. *Nat. Commun.* **15**, 1991 (2024).

30. A. S. Nicholson, D. A. Priestman, R. Antrobus, J. C. Williamson, R. Bush, S. J. McKie, H. G. Barrow, E. Smith, K. Dobrenis, N. A. Bright, F. M. Platt, J. E. Deane, Plasma membrane remodeling in GM2 gangliosidoses drives synaptic dysfunction. *PLOS Biol.* **23**, e3003265 (2025).

31. H. G. Barrow, Z. Z. Han, A. S. Nicholson, S. Strasser, D. A. Nash, J. O. Suberu, R. Antrobus, D. Te Vruchte, D. A. Priestman, S. C. Graham, F. M. Platt, J. E. Deane, Human iPSC models of ganglioside deficiency reveal a sialylated lipid requirement for plasma-membrane organization and neuronal activity. bioRxiv 2026.03.18.712603 [Preprint] (2026); https://doi.org/10.64898/2026.03.18.712603.

32. A. Fletcher-Etherington, M. P. Weekes, Quantitative temporal viromics. *Annu. Rev. Virol.* **8**, 159–181 (2021).

33. K. Nightingale, K.-M. Lin, B. J. Ravenhill, C. Davies, L. Nobre, C. A. Fielding, E. Ruckova, A. Fletcher-Etherington, L. Soday, H. Nichols, D. Sugrue, E. C. Y. Wang, P. Moreno, Y. Umrania, E. L. Huttlin, R. Antrobus, A. J. Davison, G. W. G. Wilkinson, R. J. Stanton, P. Tomasec, M. P. Weekes, High-definition analysis of host protein stability during human cytomegalovirus infection reveals antiviral factors and viral evasion mechanisms. *Cell Host Microbe* **24**, 447–460.e11 (2018).

34. S. Ozon, A. Maucuer, A. Sobel, The stathmin family--Molecular and biological characterization of novel mammalian proteins expressed in the nervous system. *Eur. J. Biochem.* **248**, 794–806 (1997).

35. I. Bièche, A. Maucuer, I. Laurendeau, S. Lachkar, A. J. Spano, A. Frankfurter, P. Lévy, V. Manceau, A. Sobel, M. Vidaud, P. A. Curmi, Expression of stathmin family genes in human tissues: Non-neural-restricted expression for SCLIP. *Genomics* **81**, 400–410 (2003).

36. Z. J. Gieroba, B.-S. Zhu, W. W. Blessing, S. L. Wesselingh, Herpes simplex virus induces Fos expression in rat brainstem neurons. *Brain Res.* **675**, 329–332 (1995).

37. B. Hu, X. Li, Y. Huo, Y. Yu, Q. Zhang, G. Chen, Y. Zhang, N. W. Fraser, D. Wu, J. Zhou, Cellular responses to HSV-1 infection are linked to specific types of alterations in the host transcriptome. *Sci. Rep.* **6**, 28075 (2016).

38. R. Hagglund, C. Van Sant, P. Lopez, B. Roizman, Herpes simplex virus 1-infected cell protein 0 contains two E3 ubiquitin ligase sites specific for different E2 ubiquitin-conjugating enzymes. *Proc. Natl. Acad. Sci. U.S.A.* **99**, 631–636 (2002).

39. C. Boutell, S. Sadis, R. D. Everett, Herpes simplex virus type 1 immediate-early protein ICP0 and is isolated RING finger domain act as ubiquitin E3 ligases in vitro. *J. Virol.* **76**, 841–850 (2002).

40. K. M. Eidson, W. E. Hobbs, B. J. Manning, P. Carlson, N. A. DeLuca, Expression of herpes simplex virus ICP0 inhibits the induction of interferon-stimulated genes by viral infection. *J. Virol.* **76**, 2180–2191 (2002).

41. T. Koshizuka, T. Kobayashi, K. Ishioka, T. Suzutani, Herpesviruses possess conserved proteins for interaction with Nedd4 family ubiquitin E3 ligases. *Sci. Rep.* **8**, 4447 (2018).

42. A. E. Cuadra, S.-H. Kuo, Y. Kawasaki, D. S. Bredt, D. M. Chetkovich, AMPA receptor synaptic targeting regulated by Stargazin interactions with the Golgi-resident PDZ protein nPIST. *J. Neurosci.* **24**, 7491–7502 (2004).

43. N. Tongmuang, M. Krishnan, V. Connor, C. Crump, L. E. Jensen, UL56 is essential for herpes simplex virus-1 virulence in vivo but is dispensable for induction of host-protective immunity. *Vaccines* **12**, 837 (2024).

44. T. Kanematsu, I.-S. Jang, T. Yamaguchi, H. Nagahama, K. Yoshimura, K. Hidaka, M. Matsuda, H. Takeuchi, Y. Misumi, K. Nakayama, T. Yamamoto, N. Akaike, M. Hirata, K.-I. Nakayama, Role of the PLC-related, catalytically inactive protein p130 in GABA(A) receptor function. *EMBO J.* **21**, 1004–1011 (2002).

45. K. Takenaka, K. Fukami, M. Otsuki, Y. Nakamura, Y. Kataoka, M. Wada, K. Tsuji, S.-I. Nishikawa, N. Yoshida, T. Takenawa, Role of phospholipase C-L2, a novel phospholipase C-like protein that lacks lipase activity, in B-cell receptor signaling. *Mol. Cell Biol.* **23**, 7329–7338 (2003).

46. E. V. Gazina, B. T. W. Leaw, K. L. Richards, V. C. Wimmer, T. H. Kim, T. D. Aumann, T. J. Featherby, L. Churilov, V. E. Hammond, C. A. Reid, S. Petrou, “Neonatal” Nav1.2 reduces neuronal excitability and affects seizure susceptibility and behaviour. *Hum. Mol. Genet.* **24**, 1457–1468 (2015).

47. K. M. Stiles, R. S. B. Milne, G. H. Cohen, R. J. Eisenberg, C. Krummenacher, The herpes simplex virus receptor nectin-1 is down-regulated after trans-interaction with glycoprotein D. *Virology* **373**, 98–111 (2008).

48. H. Tanno, T. Yamaguchi, E. Goto, S. Ishido, M. Komada, The Ankrd 13 family of UIM-bearing proteins regulates EGF receptor endocytosis from the plasma membrane. *Mol. Biol. Cell* **23**, 1343–1353 (2012).

49. Q. Zhang, M. Martin-Caraballo, S. V. Hsia, Modulation of voltage-gated sodium channel activity in human dorsal root ganglion neurons by herpesvirus quiescent infection. *J. Virol.* **94**, e01823-19 (2020).

50. N. Storey, D. Latchman, S. Bevan, Selective internalization of sodium channels in rat dorsal root ganglion neurons infected with herpes simplex virus-1. *J. Cell Biol.* **158**, 1251–1262 (2002).

51. S. G. Oakes, R. W. Petry, R. J. Ziegler, R. S. Pozos, Electrophysiological changes of HSV-1-infected dorsal root ganglia neurons in culture. *J. Neuropathol. Exp. Neurol.* **40**, 380–389 (1981).

52. J. Fukuda, T. Kurata, K. Yamaguchi, Specific reduction in Na currents after infection with herpes simplex virus in cultured mammalian nerve cells. *Brain Res.* **268**, 367–371 (1983).

53. Q. Zhang, S.-C. Hsia, M. Martin-Caraballo, Regulation of T-type Ca^{2+} channel expression by interleukin-6 in sensory-like ND7/23 cells post-herpes simplex virus (HSV-1) infection. *J. Neurochem.* **151**, 238–254 (2019).

54. Q. Zhang, S.-C. Hsia, M. Martin-Caraballo, Regulation of T-type Ca^{2+} channel expression by herpes simplex virus-1 infection in sensory-like ND7 cells. *J. Neurovirol.* **23**, 657–670 (2017).

55. R. Piacentini, D. D. Li Puma, C. Ripoli, M. Elena Marcocci, G. De Chiara, E. Garaci, A. Teresa Palamara, C. Grassi, Herpes simplex virus type-1 infection induces synaptic dysfunction in cultured cortical neurons via GSK-3 activation and intraneuronal amyloid-β protein accumulation. *Sci. Rep.* **5**, 15444 (2015).

56. F. Acuña-Hinrichsen, A. Covarrubias-Pinto, Y. Ishizuka, M. F. Stolzenbach, C. Martin, P. Salazar, M. A. Castro, C. R. Bramham, C. Otth, Herpes simplex virus type 1 neuronal infection triggers the disassembly of key structural components of dendritic spines. *Front. Cell. Neurosci.* **15**, 580717 (2021).

57. P. Bergström, E. Trybala, C. E. Eriksson, M. Johansson, T. M. Satir, S. Widéhn, S. Fruhwürth, W. Michno, F. H. Nazir, J. Hanrieder, S. R. Paludan, L. Agholme, H. Zetterberg, T. Bergström, Herpes simplex virus 1 and 2 infections during differentiation of human cortical neurons. *Viruses* **13**, 2072 (2021).

58. K. M. McCarthy, D. W. Tank, L. W. Enquist, Pseudorabies virus infection alters neuronal activity and connectivity in vitro. *PLOS Pathog.* **5**, e1000640 (2009).

59. R. Volmer, C. Monnet, D. Gonzalez-Dunia, Borna disease virus blocks potentiation of presynaptic activity through inhibition of protein kinase C signaling. *PLOS Pathog.* **2**, e19 (2006).

60. J. Gaburro, A. Bhatti, V. Sundaramoorthy, M. Dearnley, D. Green, S. Nahavandi, P. N. Paradkar, J.-B. Duchemin, Zika virus-induced hyper excitation precedes death of mouse primary neuron. *Virol. J.* **15**, 79 (2018).

61. S. Ghassemi, T. Asgari, H. Mirzapour-Delavar, S. Aliakbari, H. G. Pourbadie, C. Prehaud, M. Lafon, A. Gholami, K. Azadmanesh, N. Naderi, M. Sayyah, Lentiviral expression of rabies virus glycoprotein in the rat hippocampus strengthens synaptic plasticity. *Cell. Mol. Neurobiol.* **42**, 1429–1440 (2022).

62. P. R. Kramer, M. Umorin, R. Hornung, P. R. Kinchington, Neurexin 3α in the central amygdala has a role orofacial varicella zoster pain. *Neuroscience* **496**, 16–26 (2022).

63. M. V. Green, J. D. Raybuck, X. Zhang, M. M. Wu, S. A. Thayer, Scaling synapses in the presence of HIV. *Neurochem. Res.* **44**, 234–246 (2019).

64. J. Jędrzejewska-Szmek, D. B. Dorman, K. T. Blackwell, Making time and space for calcium control of neuron activity. *Curr. Opin. Neurobiol.* **83**, 102804 (2023).

65. Z. Li, I. P. Michael, D. Zhou, A. Nagy, J. M. Rini, Simple piggyBac transposon-based mammalian cell expression system for inducible protein production. *Proc. Natl. Acad. Sci. U.S.A.* **110**, 5004–5009 (2013).

66. R. De Pace, S. Ghosh, V. H. Ryan, M. Sohn, M. Jarnik, P. Rezvan Sangsari, N. Y. Morgan, R. K. Dale, M. E. Ward, J. S. Bonifacino, Messenger RNA transport on lysosomal vesicles maintains axonal mitochondrial homeostasis and prevents axonal degeneration. *Nat. Neurosci.* **27**, 1087–1102 (2024).

67. E. J. C. Thornburg-Suresh, D. W. Summers, Microtubules, membranes, and movement: New roles for Stathmin-2 in axon integrity. *J. Neurosci. Res.* **102**, e25382 (2024).

68. J. E. Shin, S. Geisler, A. DiAntonio, Dynamic regulation of SCG10 in regenerating axons after injury. *Exp. Neurol.* **252**, 1–11 (2014).

69. Y. Okada, H. Yamazaki, Y. Sekine-Aizawa, N. Hirokawa, The neuron-specific kinesin superfamily protein KIF1A is a unique monomeric motor for anterograde axonal transport of synaptic vesicle precursors. *Cell* **81**, 769–780 (1995).

70. M. P. Coleman, A. Höke, Programmed axon degeneration: From mouse to mechanism to medicine. *Nat. Rev. Neurosci.* **21**, 183–196 (2020).

71. Z. Melamed, J. López-Erauskin, M. W. Baughn, O. Zhang, K. Drenner, Y. Sun, F. Freyermuth, M. A. McMahon, M. S. Beccari, J. W. Artates, T. Ohkubo, M. Rodriguez, N. Lin, D. Wu, C. F. Bennett, F. Rigo, S. Da Cruz, J. Ravits, C. Lagier-Tourenne, D. W. Cleveland, Premature polyadenylation-mediated loss of stathmin-2 is a hallmark of TDP-43-dependent neurodegeneration. *Nat. Neurosci.* **22**, 180–190 (2019).

72. K. L. Krus, A. Strickland, Y. Yamada, L. Devault, R. E. Schmidt, A. J. Bloom, J. Milbrandt, A. DiAntonio, Loss of Stathmin-2, a hallmark of TDP-43-associated ALS, causes motor neuropathy. *Cell Rep.* **39**, 111001 (2022).

73. J. López-Erauskin, M. Bravo-Hernandez, M. Presa, M. W. Baughn, Z. Melamed, M. S. Beccari, A. R. A. de Almeida Quadros, O. Arnold-Garcia, A. Zuberi, K. Ling, O. Platoshyn, E. Niño-Jara, I. S. Ndayambaje, M. McAlonis-Downes, L. Cabrera, J. W. Artates, J. Ryan, A. Hermann, J. Ravits, C. F. Bennett, P. Jafar-Nejad, F. Rigo, M. Marsala, C. M. Lutz, D. W. Cleveland, C. Lagier-Tourenne, Stathmin-2 loss leads to neurofilament-dependent axonal collapse driving motor and sensory denervation. *Nat. Neurosci.* **27**, 34–47 (2024).

74. D. Freisem, D. Rombach, S. Brockmann, A. Fink, Z. Engels, A. De Luna, D. Acharya, H. Hoenigsperger, A. Goreth, S. Tigges, I. Hagmann, M. Van Gent, F. Zech, A. Ponomarenko, A. Rosenbohm, J. Dorst, S. Petri, B. Mollenhauer, J. Weishaupt, H. Tumani, M. R. Gadalla, D. Huzly, M. Gaidt, B. Sodeik, A. Viejo-Borbolla, M. Otto, T. Stamminger, F. Kirchhoff, A. Krawcyk, U. Dittmer, L. Dölken, T. Böckers, A. Catanese, G. Doblhammer, G. M. Verjans, B. B. Kaufer, M. U. Gack, F. Full, H. Hengel, V. Grozdanov, K. M. Sparrer, K. M. Danzer, Herpes simplex virus infection promotes ALS pathology through ICP0-mediated PML body

disruption. bioRxiv 2026.03.27.714707 [Preprint] (2026); https://doi.org/10.64898/2026.03.27.714707.

75. T. Koshizuka, Y. Kawaguchi, Y. Nishiyama, Herpes simplex virus type 2 membrane protein UL56 associates with the kinesin motor protein KIF1A. *J. Gen. Virol.* **86**, 527–533 (2005).

76. J. Scherer, I. B. Hogue, Z. A. Yaffe, N. S. Tanneti, B. Y. Winer, M. Vershinin, L. W. Enquist, A kinesin-3 recruitment complex facilitates axonal sorting of enveloped alpha herpesvirus capsids. *PLOS Pathog.* **16**, e1007985 (2020).

77. T. Kramer, T. M. Greco, M. P. Taylor, A. E. Ambrosini, I. M. Cristea, L. W. Enquist, Kinesin-3 mediates axonal sorting and directional transport of alphaherpesvirus particles in neurons. *Cell Host Microbe* **12**, 806–814 (2012).

78. J. Kumar, B. C. Choudhary, R. Metpally, Q. Zheng, M. L. Nonet, S. Ramanathan, D. R. Klopfenstein, S. P. Koushika, The *Caenorhabditis elegans* Kinesin-3 motor UNC-104/KIF1A is degraded upon loss of specific binding to cargo. *PLOS Genet.* **6**, e1001200 (2010).

79. J. Cisneros Solis, T. L. Blasius, K. J. Verhey, The kinesin-3 KIF1A functions as a diligent worker during axonal transport. bioRxiv 2025.09.11.675527 [Preprint] (2025); https://doi.org/10.1101/2025.09.11.675527.

80. A. Negatsch, T. C. Mettenleiter, W. Fuchs, Herpes simplex virus type 1 strain KOS carries a defective US9 and a mutated US8A gene. *J. Gen. Virol.* **92**, 167–172 (2011).

81. P. W. Howard, T. L. Howard, D. C. Johnson, Herpes simplex virus membrane proteins gE/gI and US9 act cooperatively to promote transport of capsids and glycoproteins from neuron cell bodies into initial axon segments. *J. Virol.* **87**, 403–414 (2013).

82. N. Hensel, V. Raker, B. Förthmann, A. Buch, B. Sodeik, A. Pich, P. Claus, The proteome and secretome of cortical brain cells infected with herpes simplex virus. *Front. Neurol.* **11**, 844 (2020).

83. H. T. W. Blest, A. Redmond, J. Avissar, J. Barker, A. Bridgeman, G. Fowler, L. Chauveau, J. Hertzog, I. Vendrell, R. Fischer, M. B. Iversen, L. Jing, D. M. Koelle, S. R. Paludan, B. M.

Kessler, C. M. Crump, J. Rehwinkel, HSV-1 employs UL56 to antagonize expression and function of cGAMP channels. *Cell Rep.* **43**, 114122 (2024).

84. M. X. van Bemmelen, J.-S. Rougier, B. Gavillet, F. Apothéloz, D. Daidié, M. Tateyama, I. Rivolta, M. A. Thomas, R. S. Kass, O. Staub, H. Abriel, Cardiac voltage-gated sodium channel Nav1.5 is regulated by Nedd4-2 mediated ubiquitination. *Circ. Res.* **95**, 284–291 (2004).

85. C. J. Laedermann, M. Cachemaille, G. Kirschmann, M. Pertin, R.-D. Gosselin, I. Chang, M. Albesa, C. Towne, B. L. Schneider, S. Kellenberger, H. Abriel, I. Decosterd, Dysregulation of voltage-gated sodium channels by ubiquitin ligase NEDD4-2 in neuropathic pain. *J. Clin. Invest.* **123**, 3002–3013 (2013).

86. A. García-Caballero, V. M. Gadotti, P. Stemkowski, N. Weiss, I. A. Souza, V. Hodgkinson, C. Bladen, L. Chen, J. Hamid, A. Pizzoccaro, M. Deage, A. François, E. Bourinet, G. W. Zamponi, The deubiquitinating enzyme USP5 modulates neuropathic and inflammatory pain by enhancing Cav3.2 channel activity. *Neuron* **83**, 1144–1158 (2014).

87. K. M. Wright, H. Jiang, W. Xia, M. B. Murphy, T. N. Boronina, J. N. Nwafor, H. Kim, A. M. Iheanacho, P. A. Azurmendi, R. N. Cole, P. A. Cole, S. B. Gabelli, The C-terminal of NaV1.7 is ubiquitinated by NEDD4L. *ACS Bio. Med. Chem. Au.* **3**, 516–527 (2023).

88. S. Tyagi, M.-R. Ghovanloo, M. Alsaloum, P. Effraim, G. P. Higerd-Rusli, F. Dib-Hajj, P. Zhao, S. Liu, S. G. Waxman, S. D. Dib-Hajj, Targeted ubiquitination of Na V 1.8 reduces sensory neuronal excitability. bioRxiv, 2025.02.04.636451 [Preprint] (2025); https://doi.org/10.1101/2025.02.04.636451.

89. A. B. Fotia, J. Ekberg, D. J. Adams, D. I. Cook, P. Poronnik, S. Kumar, Regulation of neuronal voltage-gated sodium channels by the ubiquitin-protein ligases Nedd4 and Nedd4-2. *J. Biol. Chem.* **279**, 28930–28935 (2004).

90. L. Qiu, X. Gao, X. Shao, J. Xi, S. Chen, T. Pham, Y. Wang, J. Dong, S. D. Rao, J. Hao, J. H. Lo, R. Yang, E. A. Engel, C. M. Crump, W. Yuan, HSV-1 UL56 protein recruits cellular NEDD4-family ubiquitin ligases to suppress CD1d expression and NKT cell function. *J. Virol.* **99**, e0214024 (2025).

91. T. Koshizuka, F. Goshima, H. Takakuwa, N. Nozawa, T. Daikoku, O. Koiwai, Y. Nishiyama, Identification and characterization of the UL56 gene product of herpes simplex virus type 2. *J. Virol.* **76**, 6718–6728 (2002).

92. Y. Ushijima, T. Koshizuka, F. Goshima, H. Kimura, Y. Nishiyama, Herpes simplex virus type 2 UL56 interacts with the ubiquitin ligase Nedd4 and increases its ubiquitination. *J. Virol.* **82**, 5220–5233 (2008).

93. H. Jiang, S. N. Thomas, Z. Chen, C. Y. Chiang, P. A. Cole, Comparative analysis of the catalytic regulation of NEDD4-1 and WWP2 ubiquitin ligases. *J. Biol. Chem.* **294**, 17421–17436 (2019).

94. M. Tao, P. C. Scacheri, J. M. Marinis, E. W. Harhaj, L. E. Matesic, D. W. Abbott, ITCH directly K63-ubiquitinates the NOD2 binding protein, RIP2, to influence inflammatory signaling pathways. *Curr. Biol.* **19**, 1255–1263 (2009).

95. U. Schubert, D. E. Ott, E. N. Chertova, R. Welker, U. Tessmer, M. F. Princiotta, J. R. Bennink, H. G. Krausslich, J. W. Yewdell, Proteasome inhibition interferes with gag polyprotein processing, release, and maturation of HIV-1 and HIV-2. *Proc. Natl. Acad. Sci. U.S.A.* **97**, 13057–13062 (2000).

96. M. Wainberg, T. Luquez, D. M. Koelle, B. Readhead, C. Johnston, M. Darvas, C. C. Funk, The viral hypothesis: How herpesviruses may contribute to Alzheimer's disease. *Mol. Psychiatry* **26**, 5476–5480 (2021).

97. G. A. Jamieson, N. J. Maitland, G. K. Wilcock, J. Craske, R. F. Itzhaki, Latent herpes simplex virus type 1 in normal and Alzheimer's disease brains. *J. Med. Virol.* **33**, 224–227 (1991).

98. Y. Dai, M. Idorn, M. C. Serrero, X. Pan, E. A. Thomsen, R. Narita, M. Maimaitili, X. Qian, M. B. Iversen, L. S. Reinert, R. K. Flygaard, M. Chen, X. Ding, B. Zhang, M. E. Carter-Timofte, Q. Lu, Z. Jiang, Y. Zhong, S. Zhang, L. Da, J. Zhu, M. Denham, P. Nissen, T. H. Mogensen, J. G. Mikkelsen, S.-Y. Zhang, J.-L. Casanova, Y. Cai, S. R. Paludan, TMEFF1 is a neuron-specific restriction factor for herpes simplex virus. *Nature* **632**, 383–389 (2024).

99. L. S. Reinert, K. Lopušná, H. Winther, C. Sun, M. K. Thomsen, R. Nandakumar, T. H. Mogensen, M. Meyer, C. Vægter, J. R. Nyengaard, K. A. Fitzgerald, S. R. Paludan, Sensing of HSV-1 by the cGAS-STING pathway in microglia orchestrates antiviral defence in the CNS. *Nat. Commun.* **7**, 13348 (2016).

100. S.-Y. Zhang, J.-L. Casanova, Genetic defects of brain immunity in childhood herpes simplex encephalitis. *Nature* **635**, 563–573 (2024).

101. T. J. Baumgartner, Z. Haghighijoo, N. A. Goode, N. M. Dvorak, P. Arman, F. Laezza, Voltage-gated Na^+ channels in Alzheimer's disease: Physiological roles and therapeutic potential. *Life* **13**, 1655 (2023).

102. J. Wang, S.-W. Ou, Y.-J. Wang, Distribution and function of voltage-gated sodium channels in the nervous system. *Channels* **11**, 534–554 (2017).

103. R. Zhao, S. D. Grunke, C. A. Wood, G. A. Perez, M. Comstock, M.-H. Li, A. K. Singh, K.-W. Park, J. L. Jankowsky, Activity disruption causes degeneration of entorhinal neurons in a mouse model of Alzheimer's circuit dysfunction. *ELife* **11**, e83813 (2022).

104. S. Meftah, J. Gan, Alzheimer's disease as a synaptopathy: Evidence for dysfunction of synapses during disease progression. *Front. Synaptic Neurosci.* **15**, 1129036 (2023).

105. A. J. Dutton, E. M. Turnbaugh, C. D. Patel, C. R. Garland, S. A. Taylor, R. Alers-Velazquez, D. M. Knipe, K. M. Nautiyal, D. A. Leib, Asymptomatic neonatal herpes simplex virus infection in mice leads to persistent CNS infection and long-term cognitive impairment. *PLOS Pathog.* **21**, e1012935 (2025).

106. L. Letenneur, K. Pérès, H. Fleury, I. Garrigue, P. Barberger-Gateau, C. Helmer, J.-M. Orgogozo, S. Gauthier, J.-F. Dartigues, Seropositivity to herpes simplex virus antibodies and risk of Alzheimer's disease: A population-based cohort study. *PLOS ONE* **3**, e3637 (2008).

107. E. Vestin, G. Boström, J. Olsson, F. Elgh, L. Lind, L. Kilander, H. Lövheim, B. Weidung, Herpes simplex viral infection doubles the risk of dementia in a contemporary cohort of older adults: A prospective study. *J. Alzheimer's Dis.* **97**, 1841–1850 (2024).

108. E. Shin, S. A. Chi, T.-Y. Chung, H. J. Kim, K. Kim, D. H. Lim, The associations of herpes simplex virus and varicella zoster virus infection with dementia: A nationwide retrospective cohort study. *Alzheimer's Res. Ther.* **16**, 57 (2024).

109. N.-S. Tzeng, C.-H. Chung, F.-H. Lin, C.-P. Chiang, C.-B. Yeh, S.-Y. Huang, R.-B. Lu, H.-A. Chang, Y.-C. Kao, H.-W. Yeh, W.-S. Chiang, Y.-C. Chou, C.-H. Tsao, Y.-F. Wu, W.-C. Chien, Anti-herpetic medications and reduced risk of dementia in patients with herpes simplex virus infections-A nationwide, population-based cohort study in Taiwan. *Neurotherapeutics* **15**, 417–429 (2018).

110. C. Schnier, J. Janbek, L. Williams, T. Wilkinson, T. M. Laursen, G. Waldemar, H. Richter, K. Kostev, R. Lathe, J. G. Haas, Antiherpetic medication and incident dementia: Observational cohort studies in four countries. *Eur. J. Neurol.* **28**, 1840–1848 (2021).

111. K. M. Prasad, S. M. Eack, M. S. Keshavan, R. H. Yolken, S. Iyengar, V. L. Nimgaonkar, Antiherpes virus-specific treatment and cognition in schizophrenia: A test-of-concept randomized double-blind placebo-controlled trial. *Schizophr. Bull.* **39**, 857–866 (2013).

112. D. P. Devanand, T. Wisniewski, Q. Razlighi, M. Qian, R. Wei, H. F. Andrews, E. P. Acosta, K. L. Bell, G. H. Pelton, D. Deliyannides, A. C. Perrin, E. Caccappolo, A. A. Gershon, K. M. Prasad, W. C. Kreisl, A. Mintz, E. D. Huey, Valacyclovir treatment of early symptomatic Alzheimer disease: The VALAD randomized clinical trial. *JAMA* **335**, 511–522 (2026).

113. M. Le Hars, C. Joussain, T. Jégu, A. L. Epstein, Non-replicative herpes simplex virus genomic and amplicon vectors for gene therapy - An update. *Gene Ther.* **32**, 173–183 (2025).

114. Y. Miyagawa, P. Marino, G. Verlengia, H. Uchida, W. F. Goins, S. Yokota, D. A. Geller, O. Yoshida, J. Mester, J. B. Cohen, J. C. Glorioso, Herpes simplex viral-vector design for efficient transduction of nonneuronal cells without cytotoxicity. *Proc. Natl. Acad. Sci. U.S.A.* **112**, E1632–E1641 (2015).

115. S. Cattaneo, B. Bettegazzi, S. Ingusci, G. Verlengia, T. A. Sofia, Z. Silvia, F. Codazzi, M. J. Morelli, M. Marzulli, J. C. Glorioso, M. Simonato, Genetic mutations in HSV-1 replication-

defective vectors: Implications for their safety in gene therapy applications. *Gene Ther.* **32**, 581–593 (2025).

116. T. Russell, B. Bleasdale, M. Hollinshead, G. Elliott, Qualitative differences in capsidless - Particles released as a by-product of bovine herpesvirus 1 and herpes simplex virus 1 infections. *J. Virol.* **92**, e01259-18 (2018).

117. B. K. Tischer, J. von Einem, B. Kaufer, N. Osterrieder, Two-step red-mediated recombination for versatile high-efficiency markerless DNA manipulation in *Escherichia coli*. *Biotechniques* **40**, 191–197 (2006).

118. P. Liljeström, S. Lusa, D. Huylebroeck, H. Garoff, In vitro mutagenesis of a full-length cDNA clone of Semliki Forest virus: The small 6,000-molecular-weight membrane protein modulates virus release. *J. Virol.* **65**, 4107–4113 (1991).

119. K. Rausalu, A. Iofik, L. Ulper, L. Karo-Astover, V. Lulla, A. Merits, Properties and use of novel replication-competent vectors based on Semliki Forest virus. *Virol. J.* **6**, 33 (2009).

120. D. A. McClelland, J. D. Aitken, D. Bhella, D. McNab, J. Mitchell, S. M. Kelly, N. C. Price, F. J. Rixon, pH Reduction as a trigger for dissociation of herpes simplex virus type 1 scaffolds. *J. Virol.* **76**, 7407–7417 (2002).

121. K. J. Livak, T. D. Schmittgen, Analysis of relative gene expression data using real-time quantitative PCR and the $2^{-\Delta\Delta CT}$ method. *Methods* **25**, 402–408 (2001).

122. S. Tyanova, T. Temu, J. Cox, The MaxQuant computational platform for mass spectrometry-based shotgun proteomics. *Nat. Protoc.* **11**, 2301–2319 (2016).

123. J. Cox, M. Mann, MaxQuant enables high peptide identification rates, individualized p.p.b.-range mass accuracies and proteome-wide protein quantification. *Nat. Biotechnol.* **26**, 1367–1372 (2008).

124. S. Tyanova, T. Temu, P. Sinitcyn, A. Carlson, M. Y. Hein, T. Geiger, M. Mann, J. Cox, The Perseus computational platform for comprehensive analysis of (prote)omics data. *Nat. Methods* **13**, 731–740 (2016).

125. V. G. Tusher, R. Tibshirani, G. Chu, Significance analysis of microarrays applied to the ionizing radiation response. *Proc. Natl. Acad. Sci. U.S.A.* **98**, 5116–5121 (2001).

126. B. T. Sherman, M. Hao, J. Qiu, X. Jiao, M. W. Baseler, H. C. Lane, T. Imamichi, W. Chang, DAVID: A web server for functional enrichment analysis and functional annotation of gene lists (2021 update). *Nucleic Acids Res.* **50**, W216–W221 (2022).

127. A. Leger, T. Leonardi, pycoQC, interactive quality control for Oxford Nanopore Sequencing. *J. Open Source Softw.* **4**, 1236 (2019).

128. H. Li, Minimap2: Pairwise alignment for nucleotide sequences. *Bioinformatics* **34**, 3094–3100 (2018).

129. R. Patro, G. Duggal, M. I. Love, R. A. Irizarry, C. Kingsford, Salmon: Fast and bias-aware quantification of transcript expression using dual-phase inference. *Nat. Methods* **14**, 417–419 (2017).

130. M. I. Love, W. Huber, S. Anders, Moderated estimation of fold change and dispersion for RNA-seq data with DESeq2. *Genome Biol.* **15**, 550 (2014).

131. V. Demichev, C. B. Messner, S. I. Vernardis, K. S. Lilley, M. Ralser, DIA-NN: Neural networks and interference correction enable deep proteome coverage in high throughput. *Nat. Methods* **17**, 41–44 (2020).

132. J. D. O'Connell, J. A. Paulo, J. J. O'Brien, S. P. Gygi, Proteome-wide evaluation of two common protein quantification methods. *J. Proteome Res.* **17**, 1934–1942 (2018).

133. S. Durinck, P. T. Spellman, E. Birney, W. Huber, Mapping identifiers for the integration of genomic datasets with the R/Bioconductor package biomaRt. *Nat. Protoc.* **4**, 1184–1191 (2009).

134. Y. Perez-Riverol, C. Bandla, D. J. Kundu, S. Kamatchinathan, J. Bai, S. Hewapathirana, N. S. John, A. Prakash, M. Walzer, S. Wang, J. A. Vizcaíno, The PRIDE database at 20 years: 2025 update. *Nucleic Acids Res.* **53**, D543–D553 (2025).

135. C. C. Friedel, A. W. Whisnant, L. Djakovic, A. J. Rutkowski, M.-S. Friedl, M. Kluge, J. C. Williamson, S. Sai, R. O. Vidal, S. Sauer, T. Hennig, A. Grothey, A. Milić, B. K. Prusty, P. J. Lehner, N. J. Matheson, F. Erhard, L. Dölken, Dissecting herpes simplex virus 1-induced host shutoff at the RNA level. *J. Virol.* **95**, e01399–20 (2021).
